# Rationale and design of randomized non-inferiority clinical trial to compare the safety and efficacy of ticagrelor monotherapy with dual antiplatelet therapy in chronic coronary syndrome patients post percutaneous coronary intervention (TICALONE-TAHA10 Protocol)

**DOI:** 10.1371/journal.pone.0325663

**Published:** 2025-07-16

**Authors:** Seyed Alireza Mirhosseini, Mohammadreza Akbari, Davar Aldavood, Hooyar Zarifkar, Armin Attar, Javad Kojuri

**Affiliations:** 1 Department of Cardiovascular Medicine, TAHA Clinical Trial Group, Shiraz University of Medical Sciences, Shiraz, Iran; 2 MD-MPH Department, School of Medicine, Shiraz University of Medical Sciences, Shiraz, Iran; 3 Shiraz Nephro-Urology Research Center, Shiraz University of Medical Sciences, Shiraz, Iran; 4 Department of Cardiology, Shiraz University of Medical Sciences, Shiraz, Iran; Campus Bio-Medico University of Rome, ITALY

## Abstract

**Background:**

Despite the wide variety of antiplatelet regimens and durations, the optimal treatment approach for chronic coronary syndrome (CCS) patients remains a subject of ongoing debate. While current guidelines recommend dual antiplatelet therapy (DAPT) with aspirin and clopidogrel, the development of drug-eluting stents (DES) and more potent agents has sparked interest in shorter DAPT regimens, followed by P2Y12 inhibitor monotherapy, as a potential alternative. Recent trials and meta-analyses have shown that this approach may provide similar protection against thrombotic events with reduced bleeding risk. Despite promising data, the safety and efficacy of Ticagrelor monotherapy specifically in CCS patients have not been rigorously tested in randomized trials.

**Methods:**

TICALONE is a non-inferiority, two-arm, double-blinded, randomized controlled clinical trial designed to evaluate the safety and efficacy of ticagrelor monotherapy compared to DAPT in CCS patients following PCI. Eligible patients undergoing PCI with drug-eluting stents will be randomly assigned to receive either conventional DAPT (aspirin and clopidogrel) or ticagrelor monotherapy for six months. Follow-up visits will be conducted at 1, 3, 6, and 12 months post-PCI to assess efficacy and safety endpoints. The primary efficacy endpoint is a composite endpoint of cardiac death, myocardial infarction, stroke, stent thrombosis, and the need for revascularization. The primary safety endpoint is the occurrence of Bleeding Academic Research Consortium (BARC) type 3 or 5 bleeding events. The secondary endpoints include components of the primary efficacy endpoint, any bleeding event (BARC type 1–5), and all-cause mortality. Ancillary endpoints are other adverse events including dyspnea, drug adherence, and reaction. All endpoints will be monitored by a Data Safety Monitoring Board (DSMB) and Trial Management Committee (TMC). Statistical analysis and reporting of trial results will follow the estimand framework. Kaplan-Meier estimates will be used to assess event rates, while the log-rank test and Cox regression analysis will be employed to compare safety and efficacy outcomes between the groups.

**Discussion:**

This trial may serve as a crucial step toward eliminating aspirin from post-PCI regimens, specifically in CCS patients. By comparing the safety and efficacy of Ticagrelor monotherapy with the conventional DAPT regimen and addressing potential risks of aspirin-free therapy and adverse events like dyspnea, this study could offer valuable insights into the possibility of P2Y12 monotherapy’s safe adoption in this population.

**Trial registration:**

TICALONE is registered at https://clinicaltrials.gov/ with the identifier NCT06509893.

## Introduction

### Background and rationale

The optimal antiplatelet therapy for patients with chronic coronary syndrome (CCS) undergoing percutaneous coronary intervention (PCI) remains uncertain. Current guidelines recommend dual antiplatelet therapy (DAPT) consisting of aspirin and clopidogrel, a P2Y12 inhibitor, for at least 6 months post-PCI, followed by antiplatelet monotherapy [1,2]. The development of antithrombotic stents, combined with the fact that ischemic risk is highest in the acute post-PCI phases and declines over time while bleeding risk remains constant, has made regimens focusing on bleeding risk more appealing. On the other hand, while DAPT reduces the risk of stent thrombosis and recurrent ischemic events, its use is associated with a significantly higher risk of bleeding compared with single antiplatelet therapy without antithrombotic benefits [3]. These considerations, along with the well-known gastrointestinal toxicity linked to aspirin, have questioned the necessity of maintaining aspirin beyond the acute post-PCI phase and made the background to develop clinical trials such as GLOBAL-LEADERS, ULTIMATE-DAPT, TWILIGHT, STOPDAPT-2, TICO and T-PASS with the aim to explore the safety and efficacy of P2Y12 inhibitor monotherapy following a short course of DAPT [4–7]. These studies have shown that shorter DAPT regimens may offer similar protection against thrombotic events while significantly reducing the risk of bleeding [8–11]. While evidence highlights the need for caution in the early discontinuation of aspirin, particularly in high-risk patients such as those with acute coronary syndrome (ACS), ASET studies have suggested that completely omitting aspirin from the regimen (aspirin-free regimen) and administering P2Y12 inhibitor (prasugrel) monotherapy following PCI is not associated with any stent thrombosis in patients with CCS [12,13]. This non-inferiority of immediate prasugrel monotherapy compared to DAPT for cardiovascular events was also demonstrated in STOPDAPT-3 [14].

Although the safety and feasibility of prasugrel monotherapy in ASET-JAPAN and ASET studies were demonstrated, STOPDAPT-3 showed that prasugrel monotherapy is not superior to DAPT for preventing bleeding in ACS or high-risk bleeding patients [13–15]. Moreover, Ticagrelor monotherapy, following 3 months of DAPT, demonstrated a 44% reduction in Bleeding Academic Research Consortium (BARC) 2–5 bleeding, with no observed difference in major adverse cardiovascular events (MACE) between the groups of high-risk patients [16]. This potent and reversible P2Y12 inhibitor, provides more consistent and rapid platelet inhibition compared to clopidogrel, resulting in a stronger anti-thrombotic effect [17]. Additionally, The PLATO trial showed a 16% reduction in MACE with Ticagrelor compared to Clopidogrel in ACS patients on a DAPT regimen, primarily due to a decrease in myocardial infarctions and cardiovascular deaths during the 1-year follow-up period [18]. On the other hand, the 90 mg twice-daily dose of ticagrelor carries a similar major bleeding risk to that of clopidogrel [19,20]. These findings suggest that ticagrelor may be a better choice for aspirin-free regimens following PCI. However, the safety and efficacy of immediate ticagrelor monotherapy in patients with CCS undergoing PCI have not yet been rigorously evaluated in randomized controlled trials.

### Objective

Building on the background presented, we designed a randomized, two-arm, double-blinded trial aims to compare the safety and efficacy of **TICA**gre**L**or m**ON**otherapy versus DAPT in CCS patients post percutaneous coronary int**E**rvention (TICALONE). We hypothesize that immediate ticagrelor monotherapy (without the DAPT period) will be non-inferior to the conventional DAPT regimen and will provide comparable protection against ischemic events while offering a reduced bleeding risk. If confirmed, this study could influence future clinical guidelines and shift the current paradigm in post-PCI management for CCS patients. The TICALONE protocol was developed through the coordination of the Prof. Kojuri Heart Clinic team and the **T**raditional and **A**dvanced **H**eart **A**pproaches clinical center, as their 10^th^ trial (TICALONE-TAHA10). It has been prepared in accordance with the guidelines outlined by the Standard Protocol Items: Recommendations for Interventional Trials (SPIRIT).

## Methods

### Study design and setting

This study is a monocentric, two-arm, double-blinded, non-inferiority, parallel, randomized, controlled clinical trial which will be conducted in Prof. Kojuri Heart Clinic located in Shiraz, southern Iran to evaluate the safety and efficacy of ticagrelor monotherapy in CCS patients following PCI. This clinic is well-suited for this research due to the high volume of CCS patients referred from primary care centers and other clinics for angiography and subsequent PCI procedures.

### Participants and eligibility criteria

Given the critical need for intensive patient control and monitoring, we have established strict inclusion and exclusion criteria. Detailed inclusion and exclusion criteria are detailed below.

Inclusion criteria:

i Male or female above 20 years of age undergoing PCI with a drug-eluting stent for chronic coronary syndrome
ii provided written informed consent, as approved by the ethics committee of the Shiraz University of Medical Sciences.

Exclusion criteria:

i Contraindication to aspirin, clopidogrel, ticagrelor, or any other reason that study drug should not be administered (including hypersensitivity, moderate or severe liver disease, active bleeding, and major surgery within 30 days)
ii Atrial fibrillation or other indication for oral anticoagulant therapy.
iii Concomitant oral or IV therapy with strong CYP3A inhibitors, CYP3A substrates with narrow therapeutic indices (cyclosporine, and quinidine), or strong CYP3A inducers (rifampin, rifampicin, phenytoin, and carbamazepine)
iv Females of childbearing age unless negative pregnancy test at screening and willing to use effective contraception for the duration of trial
v Females who are breastfeeding at the time of enrolment.
vi Unsuccessful PCI or PCI without optimal stent placement; this decision is made by the supervising interventional cardiologist.
vii Patients with planned surgical intervention to treat any cardiac or non-cardiac condition.
viii Previous PCI in the last 6 months.
ix Current (same hospitalization) or previous (within 12 months) acute coronary syndrome.
x History of definite stent thrombosis.
xi Concomitant cardiac valve disease requiring invasive therapy.
xii Acute heart failure.
xiii Active myocarditis.
xiv Cardiomyopathy.
xv Patient in hemodialysis.
xvi History of stroke or transient ischemic cerebrovascular accident.
xvii History of intracranial hemorrhage or other intracranial pathology associated with increased bleeding risk.
xviii Hemoglobin <10 g/dL
xix Peptic ulceration documented by endoscopy within the last 3 months unless healing proven by repeat endoscopy.
xx Any other condition deemed by the investigator to place the patient at excessive risk of bleeding with ticagrelor.
xxi Participation in another trial with an investigational drug or device.
xxii Assessment that the subject is not likely to comply with the study procedures or have complete follow-up.
xxiii Known drug or alcohol dependence within the past 12 months as judged by the investigator.

Given that a substantial number of patients with CCS may already be on aspirin or P2Y12 inhibitors, and to enhance the applicability of the study to real-world clinical practice for this population, prior use of these agents was not included in the exclusion criteria. However, in such cases, any ongoing aspirin or P2Y12 inhibitor therapy will be discontinued upon enrollment, and the allocated study treatment will be initiated according to the assigned group protocol.

### Intervention

**Fig 1:**
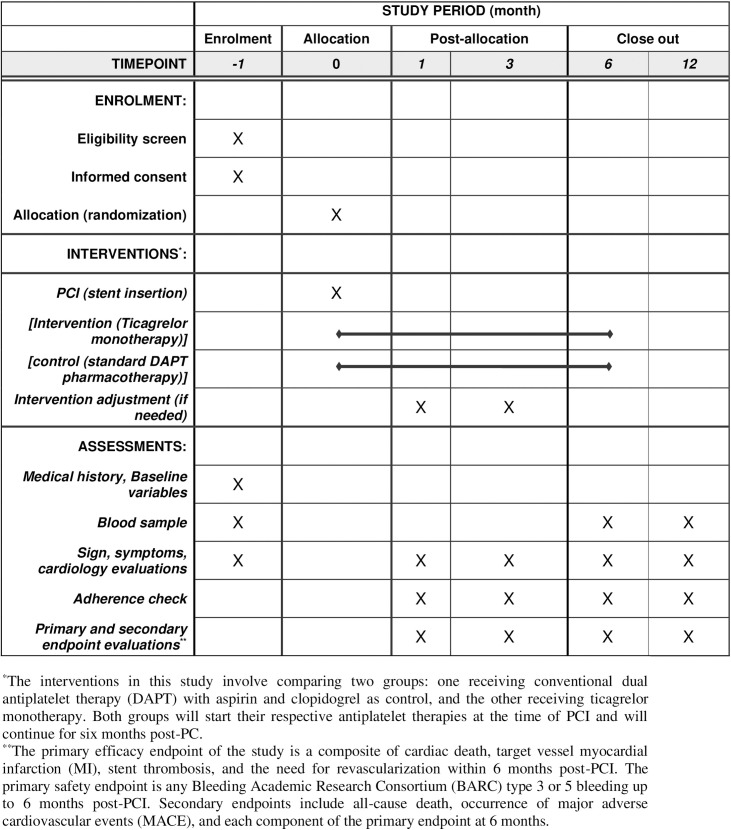
SPIRIT schedule of study timeline; TICALONE trial. *The interventions in this study involve comparing two groups: one receiving conventional dual antiplatelet therapy (DAPT) with aspirin and clopidogrel as control, and the other receiving ticagrelor monotherapy. Both groups will start their respective antiplatelet therapies at the time of PCI and will continue for six months post-PCI. **The primary efficacy endpoint of the study is a composite of cardiac death, target vessel myocardial infarction (MI), stent thrombosis, and the need for revascularization within 6 months post-PCI. The primary safety endpoint is any Bleeding Academic Research Consortium (BARC) type 3 or 5 bleeding up to 6 months post-PCI. Secondary endpoints include all-cause death, occurrence of major adverse cardiovascular events (MACE), and each component of the primary endpoint at 6 months.

An interventional cardiologist will perform angiography under the supervision of a senior interventional cardiologist. CCS patients who require revascularization based on the newest guideline [21] will undergo PCI using DES. FFR (Fractional Flow Reserve) ≤ 0.8 or iFR (Instantaneous Wave-Free Ratio) ≤ 0.89 will be considered as evidence of ischemia. PCI will be performed using the radial or femoral approach to achieve complete revascularization of at least one stenosis. All target lesions will be revascularized using the 4th generation DES. Randomization will take place after diagnostic angiography but before stent insertion (Fig 1). Eligible patients will be divided into two groups: the control group, which will get conventional DAPT with aspirin and Clopidogrel (80 mg aspirin once daily, and 75 mg clopidogrel once daily), and the experimental group, which will receive ticagrelor monotherapy (90 mg twice daily) following PCI for six months. Antiplatelet therapy will start before or at the time of PCI. Patients will receive a loading dose of assigned drugs (325 mg for aspirin, 300 mg for clopidogrel, and 180 mg for ticagrelor) before stent insertion unless they are already on pre-PCI maintenance therapy with the mentioned drugs. Peri-procedural aspirin use will not be allowed in the experimental arm.

### Modifications

Follow-up visits are scheduled at 1, 3, 6, and 12 months after the PCI procedure. If adverse events occur that require modifications to antiplatelet therapy or hypersensitivity reactions confirmed by the Data Safety and Monitoring Board (DSMB) and Trial Management Committee (TMC), drug consumption will be discontinued. These events will be recorded as study outcomes or adverse events. If the DSMB and TMC adjudicate these events as potentially leading to non-adherence to medication, the intervention group will be switched to standard care. The drugs responsible for these events will be replaced with safe and effective alternatives in both groups. Data from participants who experience such events will be censored at the time of the event to ensure accurate statistical analysis. Dyspnea, the most common side effect of ticagrelor, will be carefully monitored and recorded during follow-up visits. In case of severe dyspnea which may lead to non-adherence to the medication, the patient will be switched from the experimental therapy to the standard regimen. Non-severe dyspnea will be managed using conservative strategies like recommending caffein, theophylline, or inhaled bronchodilator in more severe cases.

### Adherence

To enhance treatment adherence, the cardiologist will emphasize the importance of taking the study medication as prescribed, including the correct dose, timing, and drug characteristics, during each visit. Patients will also receive detailed instructions about the purpose, use, and benefits of the procedures and pharmacotherapy involved in the study. The cardiologist will remind participants of the importance of contacting the clinic if they experience any issues potentially related to the study, such as bleeding, chest pain, cold sweats, or other related symptoms. Additionally, patients will be asked about any difficulties they may have in taking their study medication.

### Concomitant care

The administration of any antiplatelet or anticoagulant other than study pills is prohibited. The prescription of additional medications for the prevention or treatment of other conditions, according to current guidelines, is permitted only if there is no interaction with the study medications. If a patient requires anticoagulant therapy that interferes with the study drugs due to a specific illness or event, the event will be recorded, and the patient will be censored. At each follow-up visit, patients will be asked about any new medications, illnesses, signs, or symptoms they experience during the study. Medication adjustments will be made as necessary to prevent any impact on the trial results.

### Outcomes

The co-primary efficacy endpoint is a composite of cardiac death, MI, stroke, stent thrombosis, and the need for revascularization. The secondary endpoints are all-cause death, occurrence of MACE including cardiac death, stroke (ischemic, hemorrhagic, or unknown), MI, arrhythmia, and other component of the co-primary endpoint. The primary safety endpoint is any Bleeding Academic Research Consortium (BARC) bleeding type 3 or 5 after PCI. The secondary safety endpoint is the occurrence of BARC type 1–5 bleeding. Ancillary endpoints are other adverse events including dyspnea, drug adherence, and reaction. All endpoints will be evaluated in each follow-up visit and the results of the analysis of endpoints will be reported at the end of 6- and 12- months post PCI. All events will be adjudicated by DSMB and TMC.

### Participant timeline

**Fig 2:**
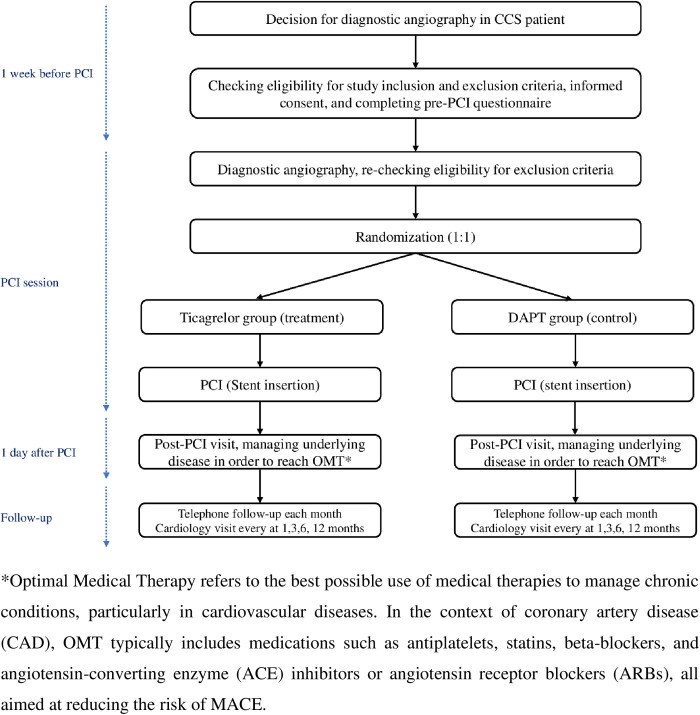
The flow diagram of the study. *Optimal Medical Therapy refers to the best possible use of medical therapies to manage chronic conditions, particularly in cardiovascular diseases. In the context of coronary artery disease (CAD), OMT typically includes medications such as antiplatelets, statins, beta-blockers, and angiotensin-converting enzyme (ACE) inhibitors or angiotensin receptor blockers (ARBs), all aimed at reducing the risk of MACE.

After a clear decision to conduct diagnostic angiography for CCS patients based on current guidelines [22,23]. They will be informed about the study and available options. After providing the informed consent, the pre-PCI questionnaire will be completed by an interventional cardiologist and a member of TMC. Patients will be randomized to treatment or control group after diagnostic angiography and before stent insertion. The loading dose of the assigned medications for each group will be administered to the patient prior to stent insertion. After the procedure, the enrolled patients will be visited for evaluation and management of underlying diseases, routine assessments, and optimal medical therapy. Clinical follow-ups will be performed at 1, 3, 6, and 12 months and telephone contacts will be performed every month till the end of the study. An assessment of angina status, cardiovascular drug use, dyspnea, and any serious adverse events will be recorded during these clinical follow-up visits. Illustrative participant timeline is shown in Figs 1 and 2.

### Sample size

This study is designed to compare 6- and 12-month ticagrelor monotherapy to the conventional DAPT treatment in terms of efficacy and safety. We hypothesize that ticagrelor monotherapy is non-inferior for safety and efficacy to the DAPT regimen. The null hypothesis is the superiority of the standard DAPT regimen for both safety and efficacy primary endpoint. Based on the results of the international trials (GLOBAL LEADERS, STOP-DAPT-2, and TICO trials) and related article in our country (19) we anticipate that 2% of patients in the control and treatment arm will reach the co-primary efficacy endpoint. The non-inferiority margin is assumed to be 50% of the mentioned cumulative incidence (1%). The level of significance (one-sided alpha) and power (1-β) of the study were considered 5% and 80% respectively. Based on these assumptions, 2424 patients are required for each arm of the ideal study. Taking into consideration of loss to follow-up cases (10%), we have to enroll 5400 patients with 1:1 randomization to each arm in the ideal study. The formula that has been used for these calculations is shown in Formula 1 (20). However, due to the limited number of cases referred to our center and the safety uncertainties of the project, we will first conduct the initial phase of the study. We estimated that a total of 400 patients would be referred to our center for CCS management over a three-month period (the expected recruitment time for initial phase). With the goal of reaching the target sample size within approximately three years, the suitable sample size for the initiation phase is calculated to be about 180 patients in each arm of the study using Formula 2.

**Fomula 1**: $\backslash (N=2\;\times \;{\left(\frac{Z_{1-\alpha }+Z_{1-\beta }}{\delta_{0}}\right)}^{2}\times p\;\times (1-p).\;\backslash )$ N = size per group; p = the response rate of standard treatment group; z(x)= the standard normal deviate for a one or two sided x; δ0 = a clinically acceptable margin

**Fomula 2**: $\backslash (n=\;\frac{n_{0}}{1+\;\frac{\left(n_{0}-1\right)}{N}}\;\backslash )$. n=suitable sample size for the study; n0=ideal sample size x; N= total patients available

### Recruitment

Each patient referring to our center for CCS management is observed and tested for diagnostic angiography indications. On average, in our center, 100–200 CCS patients undergo PCI monthly. In addition to the relatively large number of patients referred to our clinic, they typically come from a high socioeconomic background, which helps ensure better compliance and adherence to the prescribed medications.

### Randomization (sequence generation, concealment, implementation)

Participants will be randomly allocated to either the control group or the experimental group in a 1:1 ratio using permuted block randomization through a web-based randomization service (https://www.sealedenvelope.com/randomisation/simulation/). Allocation concealment will be ensured as the randomization service will not be accessed by the interventional cardiologist until the patient has been recruited into the trial. Recruitment occurs after diagnostic angiography and before stent insertion. The only person who is involved in randomization is the interventional cardiologist who conducts PCI.

### Blinding

The allocation of patients to each group is known only to the cardiologist responsible for randomization. This cardiologist is not involved in the patient’s treatment, follow-up, outcome assessment, or data analysis to ensure blinding of the physician team to the allocation. The allocation is stored using a code number in a computer-based data system, which will remain blinded until the analysis is complete. Neither the patients, investigators, nor data analysts will be aware of the group assignment. To ensure the blinding of participants and prevent them from identifying their assigned treatment group, despite differences in the type and number of medications, a standardized method of tablet administration was employed. Following randomization and prior to stent insertion, all patients will receive two unmarked tablets as a loading dose. In the control group, patients will be administered one 325 mg aspirin tablet and one 300 mg clopidogrel tablet. In contrast, patients in the intervention group will receive two 90 mg ticagrelor tablets, administered by the cardiologist responsible for the randomization process. For the maintenance dose, each patient will be provided with two boxes of tablets and instructed to take one tablet from each box daily, one in the morning (8:00 AM) and one in the evening (8:00 PM). In the intervention group, both boxes will contain 90 mg ticagrelor tablets, whereas in the control group, one box will contain 80 mg ASA tablets and the other will contain 75 mg clopidogrel tablets. The same cardiologist responsible for randomization will also distribute the medication boxes containing the required drugs for the six-month follow-up period. This approach is designed to preserve the double-blind nature of the study, ensuring that participants, physicians, outcome assessors, and other study personnel remain unaware of group allocations. In the event of adverse reactions or if a medication switch is deemed necessary by the DSMB or TMC, the group allocation will be unblinded, and appropriate adjustments will be made.

### Data collection and management

After the procedure, the enrolled patients will be visited for evaluation and management of underlying diseases, routine assessments, and optimal medical therapy. Clinical follow-ups will be performed at 1, 3, 6, and 12 months and telephone contacts will be performed every month till the end of the study. An assessment of angina status, cardiovascular drug use, and any serious adverse events will be recorded during these clinical follow-up visits. Participant files are to be stored in numerical order and stored in a secure and accessible place and manner. Files will be scanned and data will be recorded in computer-based data system used for patient data gathering and archiving. This program is well encrypted.

### Statistical analysis

Continuous variables will be presented as mean ± SD and categorical variables will be presented as counts and percentages. The comparison of categorical variables will be conducted using either the χ² test or Fisher’s exact test. To compare continuous variables, the Student’s t-test or the Wilcoxon rank-sum test will be used for non-normally distributed data. The statistical analyses will be carried out using Stata version 18.0 (StataCorp LLC, College Station, Texas, USA). All primary and secondary endpoints will be analyzed both on an intention-to-treat basis, determined by allocation. Cumulative event rates will be estimated with the Kaplan-Meier method. The Cox log-rank approach will be utilized to compare the incidence of endpoints, specifically focusing on the time-to-first-event analyses. Patients lost to follow-up will be censored at the time of the last known contact. The null hypothesis will be evaluated on the intention-to-treat population using inferiority statistics. If the upper limit of the one-sided 95% confidence interval of the difference is less than the prespecified non-inferiority margin of 1%, ticagrelor monotherapy will be considered non-inferior to the conventional 6-month DAPT regimen. A subgroup analysis will be conducted for clinically relevant factors such as age, sex, comorbidities, prior use of aspirin or P2Y12 inhibitors, lesion or procedural characteristics, and other risk indicators. A two-sided test will be used for all hypotheses testing at a 5% significance level. The results of this study will be reported in accordance with CONSORT statement. Statistical analysis and reporting of trial results will follow the estimand framework [24].

### Treatment of missing values

The primary analysis of the study endpoints will not be covariate-adjusted. No imputation methods will be used to infer missing values of baseline variables. For the study endpoints, we will censor patients lost to follow-up and regard them as not having the primary endpoint when estimating Kaplan–Meier event rates. If a medication switch occurs, the patient will be considered to have reached the study endpoint at the time of the switch, and the remaining time in the study will not be included in further analysis.

### Data monitoring, harm, and auditing

Prior to conducting statistical analysis, an expert from the cardiology department, who is not part of the research team, will review and validate all measurements. This adjudicator will examine the quality of each measurement and will omit any that do not meet the required standards, treating them as missing data in the analysis. Additionally, an impartial safety committee, working without knowledge of the treatment assignments, will review and determine any significant MACE. After this adjudication process is finalized, the database will be made accessible for data analysts. The study’s executive committee will communicate any adverse events to a separate DSMB. This board, which operates independently, holds the power to halt the trial prematurely in cases where patient safety is at risk or the main research goal is achieved. If the adverse event rate (primary safety and efficacy endpoint) in the intervention group surpasses that of the control group by more than 20% during any interim analysis, the DSMB will review the data and may recommend termination of the trial if necessary. The DSMB will be responsible for regular safety monitoring, including unexpected serious adverse events, mortality, and severe bleeding, with safety reports generated for review. Additionally, all MACE and bleeding events will be promptly reported to the DSMB, which will conduct independent audits each month. In addition to tracking MACE and bleeding, we will systematically collect data on other potential harms, such as drug reactions, dyspnea, and adverse events, through both spontaneous reporting and structured follow-up visits. Adverse events will be recorded, analyzed, and reported using Common Terminology Criteria for Adverse Events version 4.0 (CTCAE v4.0). All harms, regardless of their frequency, will be reported in publications to provide a comprehensive view of the safety profile. This approach ensures transparency and provides a detailed understanding of the potential risks associated with the interventions.

### Ethical considerations

This protocol and the informed consent form are approved by the Institutional Review Boards/Ethical Committee of Shiraz University of Medical Sciences on June 01, 2024, under the ethics code IR.SUMS.MED.REC.1403.150. we registered TICALONE-TAHA10 at https://www.ClinicalTrials.gov with the trial identifier NCT06509893 and https://www.irct.ir (Iranian Registry of Clinical Trials) under the trial identifier IRCT20240701062299N1.

### Protocol amendments

Any modifications to the protocol which may impact on the conduct of the study, potential benefit of the patient or may affect patient safety, including changes of study objectives, study design, patient population, sample sizes, study procedures, or significant administrative aspects will require a formal amendment to the protocol applied by TMC. Such amendment will be approved by the Ethics Committee/Institutional Review Board of Shiraz University of Medical Sciences.

### Consent or assent

The interventional cardiologist will introduce the trial to patients. Patients will also receive information sheets. Patients will then be able to have an informed discussion with the participating consultant. A trained general practitioner will obtain written consent from patients willing to participate in the trial.

### Ancillary and post-trial care

Ancillary study will not be conducted on data derived from this trial. Shiraz University of Medical Sciences will provide insurance coverage for any harm that participants may experience as a result of the study protocol. This insurance will cover additional healthcare costs, compensation, or damages directly linked to the study procedures.

### Access to data

Only members of the TMC will have access to patient data and files.

### Confidentiality

All study-related information will be stored securely at the study program (computer-based). All participant information will be stored in locked file cabinets in areas with limited access. All laboratory specimens, reports, data collection, process, and administrative forms will be identified by a coded ID to maintain participant confidentiality. All records that contain names or other personal identifiers, such as locator forms and informed consent forms, will be stored separately from study records identified by code number. All local databases will be secured with password-protected access systems.

## Discussion

The GLOBAL-LEADERS trial was the first study to clinically evaluate P2Y12 monotherapy followed by a short period of DAPT, comparing 1 month of DAPT followed by 23 months of ticagrelor monotherapy against the standard regimen of 12 months of DAPT followed by aspirin. At the 24-month follow-up, there was no significant difference in the primary ischemic endpoint of all-cause death and MI between the two groups. Importantly, no safety concerns, such as increased bleeding, were noted [25]. Following GLOBAL-LEADERS, some randomized controlled trials (RCTs) consistently demonstrated that discontinuing aspirin after 1−3 months and continuing P2Y12 inhibitor monotherapy significantly reduced bleeding without an increase in thrombotic events [9,11,16,26]. These findings, along with the results from other trials such as TWILIGHT, have contributed to pooled analyses showing that a very short course of DAPT followed by P2Y12 inhibitor monotherapy significantly reduces bleeding, including major bleeding, without compromising ischemic protection [3–5]. Recent individual patient data (IPD)-level meta-analysis of randomized trials have shown that DAPT de-escalation to ticagrelor monotherapy after 2 week to 3 months of DAPT post PCI is non-inferior for MACE (HR 0·91 [95% CI 0·78–1·07]; p = 0·0039 for non-inferiority; τ2 < 0·0001), and superior for bleeding events (HR 0·43 [95% CI 0·34–0·54]; p < 0·0001 for superiority; τ2 = 0·079) compared to 12-month DAPT regimen in ACS patients [27]. Another IPD-level meta-analysis indicated that both clopidogrel and ticagrelor monotherapy after short term DAPT is associated with reduced bleeding but only ticagrelor monotherapy was non inferior to DAPT for MACE in patients undergoing PCI [28]. These results support the growing evidence that paves the way for aspirin-free therapy using the suitable P2Y12 inhibitor. The TICALONE trial represents another step toward replacing DAPT with P2Y12 monotherapy by omitting the aspirin immediately from post-PCI regimen. Recognizing the potential risk of adverse events, if aspirin-free therapy is initiated immediately after PCI, particularly in high-risk patients [29], we designed this trial to exclusively include patients with CCS. This is another key distinguishing feature of the TICALONE trial, as it specifically focuses on evaluating patients with CCS, unlike previous trials that included a mix of both ACS and CCS patients.

The fact that P2Y12 monotherapy have superior efficacy and similar safety compared to aspirin monotherapy align with the current guidelines recommendations that prioritize the discontinuation of aspirin to discontinuation of P2Y12 inhibitor [21,30]. Choosing the appropriate P2Y12 inhibitor as an alternative to DAPT required a careful evaluation of the benefits and side effects of each drug. A previous meta-analysis revealed no significant difference between ticagrelor and Prasugrel in their effects on ACS patients [31]. However, research on prasugrel is more limited, and it is less widely available in global markets. The mentioned meta-analysis also demonstrated that, compared to clopidogrel, ticagrelor significantly reduced both all-cause mortality and CV deaths. We recognize that a major drawback of ticagrelor is reduced treatment compliance due to the dyspnea it can cause. To address this, we will closely monitor patients during follow-up visits to assess any occurrences of dyspnea and will report its prevalence among study participants.

The elimination of aspirin as the backbone of current regimens may lead to unforeseen adverse events and complications. For this reason, we designed the TICALONE trial to begin with an initiation phase involving 360 participants, followed by a 6- and 12-month follow-up. After releasing the initial results, we will evaluate whether to continue the trial as planned to reach the ultimate sample size or to make adjustments to the trial design. This approach allows us to refine the study in order to achieve the best outcomes and contribute to determining the most appropriate post-PCI regimen for CCS patients. We designed this trial with a 12-month follow-up, but longer-term evaluation and assessment of CCS patients are necessary to determine the most suitable regimen after PCI. We look forward to the initial results, which will guide the continuation of the study and help the TICALONE team develop the most accurate recommendations for updating clinical guidelines.

We acknowledge the single-center design of the study as a limitation, but our center is recognized as a referral clinic for CCS patients from southern Iran, and we have an established electronic database that facilitates the enrollment process, data collection, and patient follow-up. Additionally, educational initiatives provided by our staff and physicians help enhance patient adherence to treatment and compliance with the study objectives. Additionally, as the first trial to evaluate immediate ticagrelor monotherapy in patients with coronary syndrome, we applied strict inclusion and exclusion criteria to ensure an appropriate study design for obtaining reliable results. While this approach may limit the generalizability of the findings, it can be addressed through further secondary analyses.

TICALONE may serve as a critical bridge in testing the feasibility of replacing DAPT with P2Y12 monotherapy, at least for low-risk patients, as it is specifically designed to evaluate CCS patients. This trial could provide key insights into whether P2Y12 monotherapy can effectively and safely be used in this population.

## Dissemination policy

### Trial results

The trial results will be published after 6 and 12 months of follow-up, first as short-term results and later as long-term results. The findings will be shared with participating physicians, referring physicians, patients, and the broader medical community as soon as possible after analysis.

### Authorship

Authorship will be discussed and determined in TMC

### Reproducible research

Full protocol of the study will be publicly available as soon as possible.

### Appendices

Appendix 1in S1 Data shows the variables and components of the baseline medical evaluation of the enrolled patients. Follow-up variables are detailed in Appendix 2 in S1 Data. Informed consent materials are presented in Appendix 3 in S1 Data.

### Biological specimens

Blood samples will be collected to perform a complete blood count (CBC) and to measure blood urea nitrogen (BUN) and creatinine (Cr) levels. Additionally, serum levels of partial thromboplastin time (PTT), prothrombin time (PT), and international normalized ratio (INR) will be analyzed.

### Table of execution timelines

See Fig 1.

### Trial status

After completing the trial protocol and registering the study on ClinicalTrials.gov, recruitment began on August 1, 2024. We expect recruitment to conclude by December 1, 2024. Following a 6-month follow-up period, the first results of the study are projected to be published in the first half of 2025.

### Protocol amendment

This version is the second revision of the protocol completed on September 1, 2024.

## Supporting information

S1 Data Supplementary Material Regarding TICALONE-TAHA10 Study Protocol Appendix 1 - Baseline Characteristics
Appendix 2 - Follow-up Variables
Appendix 3 - Informed Consent Form
Ethics Approval Funding Contract
SPRITI checklist.
(ZIP)

## Abbreviations

CCS: Chronic Coronary Syndrome
PCI: Percutaneous Coronary Intervention
DAPT: Dual Antiplatelet Therapy
DES: Drug-Eluting Stent
BARC: Bleeding Academic Research Consortium
MACE: Major Adverse Cardiovascular Events
ACS: Acute Coronary Syndrome
SC: Steering Committee
TMC: Trial Management Committee
DSMB: Data Safety Monitoring Board
SPIRIT: Standard Protocol Items, Recommendations for Interventional Trials
OMT: Optimal Medical Therapy
INR: International Normalized Ratio
CBC: Complete Blood Count
BUN: Blood Urea Nitrogen
PT: Prothrombin Time
PTT: Partial Thromboplastin Time
AHA: American Heart Association
ACC: American College of Cardiology
ESC: European Society of Cardiology
RCT: Randomized Controlled Trial
