# Supplementary material for: Rationale and design of randomized non-inferiority clinical trial to compare the safety and efficacy of ticagrelor monotherapy with dual antiplatelet therapy in chronic coronary syndrome patients post percutaneous coronary intervention (TICALONE-TAHA10 Protocol)
Source: PLoS One. 2025 Jul 16;20(7):e0325663. doi: 10.1371/journal.pone.0325663 (PMC12266445; doi:10.1371/journal.pone.0325663)
Supplement: S1 Data — Appendix 1 - Baseline Characteristics Appendix 2 - Follow-up Variables Appendix 3 - Informed Consent Form Ethics Approval Funding Contract SPRITI checklist. (ZIP) [file pone.0325663.s001.zip › supporting data/Appendix 1 - baseline data[1].docx]

Data gathering – TICALONE project – appendix1

Baseline data

1. Demographics

- Age

- Gender

- Weight

- Height

- Body Mass Index (BMI)

2. Past Medical History

- History of coronary artery disease (CAD)

- Previous myocardial infarction (MI)

- History of heart failure

- NYHA class

- History of hypertension

- History of diabetes mellitus

- History of hyperlipidemia

- Previous percutaneous coronary intervention (PCI) or coronary artery bypass grafting (CABG)

- History of stroke or transient ischemic attack (TIA)

- History of peripheral artery disease

- History of chronic kidney disease

- History of liver disease

- History of bleeding disorders

3. Past Surgical History

- Prior cardiac surgeries (e.g., CABG)

4. Social History

- Smoking status (current, former, never)

- Alcohol consumption (amount and frequency)

- Drug use (recreational drugs)

5. Drug History

- Current medications (including dosages and duration)

- Previous use of antiplatelet agents (aspirin, clopidogrel, ticagrelor, prasugrel, etc.)

- Use of anticoagulants (warfarin, direct oral anticoagulants)

- Use of antihypertensives (beta-blockers, ACE inhibitors, ARBs, calcium channel blockers, diuretics)

- Use of statins or other lipid-lowering agents

- Use of antidiabetic medications (insulin, oral hypoglycemics)

- Use of other cardiovascular medications (nitrates, antiarrhythmics)

- Known drug allergies or adverse reactions

6. Baseline Echocardiographic Findings

- Left ventricular ejection fraction (LVEF)

- Wall motion abnormalities

- Presence of left ventricular hypertrophy

- Valvular function (stenosis or regurgitation)

- Presence of intracardiac thrombus

- Presence of PE

- Pericardial effusion

7. Baseline ECG Findings

- Heart rate (brady- or tachycardia)

- Rhythm (sinus rhythm, atrial fibrillation, etc.)

- Presence of ischemic changes (ST-segment elevation or depression, T-wave inversions)

- Presence of conduction abnormalities (bundle branch blocks, AV blocks)

- Presence of pathological Q waves

- Any arrhythmias noted

8. Baseline Laboratory Data

- Complete Blood Count (CBC)

- Hemoglobin

- Hematocrit

- White blood cell count

- Platelet count

- Coagulation profile

- Prothrombin Time (PT)

- Partial Thromboplastin Time (PTT)

- International Normalized Ratio (INR)

- Renal function tests

- Blood Urea Nitrogen (BUN)

- Serum Creatinine

- Estimated Glomerular Filtration Rate (eGFR)

- Lipid profile

- Total Cholesterol

- Low-Density Lipoprotein (LDL)

- High-Density Lipoprotein (HDL)

- Triglycerides

- Blood glucose levels

- Fasting blood glucose
