# Supplementary material for: Rationale and design of randomized non-inferiority clinical trial to compare the safety and efficacy of ticagrelor monotherapy with dual antiplatelet therapy in chronic coronary syndrome patients post percutaneous coronary intervention (TICALONE-TAHA10 Protocol)
Source: PLoS One. 2025 Jul 16;20(7):e0325663. doi: 10.1371/journal.pone.0325663 (PMC12266445; doi:10.1371/journal.pone.0325663)
Supplement: S1 Data — Appendix 1 - Baseline Characteristics Appendix 2 - Follow-up Variables Appendix 3 - Informed Consent Form Ethics Approval Funding Contract SPRITI checklist. (ZIP) [file pone.0325663.s001.zip › supporting data/Appendix 2 - Follow up data[1].docx]

Follow-up variables and definitions

Death

Presence/reason/days from PCI

Cardiovascular Death

Presence/days from PCI

Need for revascularization

Presence/days from PCI

Target vessel MI

Presence/days from PCI

Non-target vessel MI

Presence/days from PCI

Stent thrombosis (confirmed by repeat PCI)

Presence/days from PCI

Stroke (ischemic or hemorrhagic)

Presence/type/days from PCI

Hospitalization due to cardiovascular disease

Presence/diagnosis/days from PCI

Bleeding and BARC-type

Presence/BARC-type/days from PCI

BARC types are defined and explained in a separate file

Follow-up data (at 1, 3, 6, and 12 months post PCI)

Clinical Assessments

- **Symptom Assessment**

- Presence and severity of angina (using Canadian Cardiovascular Society grading)

- Dyspnea

- Fatigue

- Palpitations

- Syncope

- **Vital Signs**

- Blood pressure (SBP and DBP)

- Heart rate

- **Medication Adherence**

- Compliance with antiplatelet therapy (ticagrelor or DAPT)

- Adherence to other prescribed medications

- **Adverse Events**

- Any bleeding events (categorized by BARC type)

- Any other side effects or adverse reactions

- Hospitalizations since the last visit

- **Interventions**

- Any additional interventions or procedures since the last visit (e.g., repeat PCI, CABG)

Final follow-up (at 1, 3, 6, and 12 months post PCI)

All of above data should gather plus:

Laboratory Tests

- **Complete Blood Count (CBC)**

- Hemoglobin

- White blood cell count

- Platelet count

- **Coagulation Profile**

- Prothrombin Time (PT)

- Partial Thromboplastin Time (PTT)

- International Normalized Ratio (INR)

- **Renal Function**

- Serum Creatinine

- eGFR

- **Lipid Profile**

- Total Cholesterol

- Low-Density Lipoprotein (LDL)

- High-Density Lipoprotein (HDL)

- Triglycerides

- **Glucose Levels**

- Fasting blood glucose

- **Inflammatory Markers**

- C-Reactive Protein (CRP)

Diagnostic Tests

- **Electrocardiogram (ECG)**

- Rhythm

- Presence of ischemic changes

- New or persistent conduction abnormalities

- **Echocardiogram (if indicated)**

- Left ventricular ejection fraction (LVEF)

- Wall motion abnormalities

- Valvular function
