# Supplementary material for: Rationale and design of randomized non-inferiority clinical trial to compare the safety and efficacy of ticagrelor monotherapy with dual antiplatelet therapy in chronic coronary syndrome patients post percutaneous coronary intervention (TICALONE-TAHA10 Protocol)
Source: PLoS One. 2025 Jul 16;20(7):e0325663. doi: 10.1371/journal.pone.0325663 (PMC12266445; doi:10.1371/journal.pone.0325663)
Supplement: S1 Data — Appendix 1 - Baseline Characteristics Appendix 2 - Follow-up Variables Appendix 3 - Informed Consent Form Ethics Approval Funding Contract SPRITI checklist. (ZIP) [file pone.0325663.s001.zip › supporting data/Appendix 3 - informed consent materials[1].pdf]

In the Name of God

Informed Consent Form

Participation in the Research Project

Title of the Research Project:

A Comparative Study on the Safety and Efficacy of Ticagrelor Monotherapy Versus Standard Dual Antiplatelet Therapy in Patients with Chronic Coronary Syndrome Post Percutaneous Coronary Intervention (PCI); A Six-Month, Single-Center, Randomized Clinical Trial

Project Number:

29781

Principal Investigators:

Dr. Javad Kojuri, Dr. Davar Al Davood Zoleid

Affiliated Faculty or Unit:

Faculty of Medicine, Shiraz University of Medical Sciences, Department of Cardiology, Professor Kojuri Heart Specialty Clinic

Objectives of the Research:

To evaluate the safety and efficacy of Ticagrelor compared to a combination of aspirin and clopidogrel in patients with chronic chest pain post-PCI (stent placement).

Participation Procedure:

Patients participating in this study will be randomly assigned to two groups: one receiving Ticagrelor monotherapy and the other receiving aspirin and clopidogrel combination therapy after stent placement. Informed consent will be obtained prior to the study. Blood samples will be collected, and patient characteristics will be fully evaluated. Following the intervention, monthly evaluations will be conducted via telephone, with in-person assessments every two months for six months.

Blood Sampling:

Blood samples will be taken once before the intervention and once after six months to test blood parameters, coagulation profiles, lipid profiles, and electrolytes.

Potential Benefits:

A potential benefit of this study is reducing bleeding risks associated with aspirin use. Patient participation in this study may also contribute to improving future diagnostic and therapeutic methods.

Potential Risks:

Stent thrombosis, myocardial infarction, need for revascularization, and bleeding.

Risk Mitigation:

Patients will be closely monitored through regular visits during the six-month study, and any necessary interventions will be promptly carried out to minimize potential risks.

Costs:

The costs involved in this study are covered by Shiraz University of Medical Sciences.

Alternative Options for Participation in the Study:

If the patient declines to participate in the study, routine treatment with aspirin and clopidogrel will be administered post-intervention.

Confidentiality:

Test results and study information will be kept confidential and only shared privately with the patient. Investigators will remain blinded to patient data post-intervention, knowing only the medication used without patient identification. The Shiraz University of Medical Sciences Ethics Committee may access patient information to ensure participant rights are respected. Informed consent is required for using collected samples in future research.

Responding to Questions:

Patients can direct any questions or concerns to the office of the Principal Investigator (Dr. Kojuri), located at Chamran Blvd., Niayesh Building, Shiraz, with the contact number 07136540068.

Right to Refuse or Withdraw:

Participation in this study is entirely voluntary, and I am free to refuse participation or withdraw at any time without affecting my medical care or treatment.

Feedback, Suggestions, and Problem Reporting:

Thank you for your cooperation in this research project. Please share any comments, suggestions, or issues you encounter during the study by contacting the Shiraz University of Medical Sciences Ethics Committee at 32122389 or 32122438, or via email at researchethic@sums.ac.ir, or fax at 32122686. Any concerns will be confidentially addressed by the committee.

Clinical Trial Registration:

Dear Participant, you can learn more about this study by visiting the Iranian Clinical Trial Registry at www.irct.ir. The study registration code is as follows:

Clinical Trial Registration Code: IRCT20240701062299N1

Consent

I, ____________, give my informed consent to participate in this study. I understand that all personal information will remain confidential, and the results will be presented anonymously as part of group data. Any individual results will be reported without revealing my identity if necessary. This agreement does not prevent me from taking legal action against (name of unit) in the event of unethical or illegal conduct.

Signature and Fingerprint of Participant:

Witness's Name, Contact Number, and Signature:

Researcher's Name and Signature:

Contact Number:

Mobile Number:

Date:

Notes:

This form must be produced in three copies: one for secure storage for monitoring by ethics committees, one for the participant, and one to be attached to the patient’s medical record. The right index finger's fingerprint is required; if unavailable, an explanation should be provided. The legal age for consent is 18 years and above. Vulnerable groups, including children, pregnant women, people with mental disabilities, prisoners, and emergency patients, require consent from a legal guardian.

University Ethics Committee in Medical Research - Shiraz University of Medical Sciences

کمیته دانشگاهی اخلاق در پژوهش های علوم پزشکی
دانشگاه علوم پزشکی شیراز

باسمه تعالی

فرم رضایت آگاهانه

شرکت در طرح پژوهشی

**عنوان طرح پژوهشی**	مقایسه ایمنی و اثربخشی تک درمانی تیکاگرلور در مقایسه با درمان ضد پلاکتی دوگانه استاندارد در بیماران مبتلا به سندرم کرونر مزمن پس از مداخله از طریق پوست(PCI)؛ یک کار آزمایی بالینی تصادفی تک مرکزی شش ماهه
**شماره طرح پژوهشی**	29781
**نام مجری یا مجریان**	دکتر جواد کجوری ، دکتر داور آل داود ذوالید
**دانشکده یا واحد مربوطه**	دانشکده پزشکی دانشگاه علوم پزشکی شیراز – بخش قلب و عروق – کلینیک فوق تخصصی قلب استاد پروفسور کجوری
**اهداف پژوهش**	بررسی میزان اثربخشی و ایمنی استفاده از داروی تیکاگرول در مقایسه با ترکیب آسپیرین و کلوئیدوگرل برای بیماران درد قفسه سینه مزمن بعد از انجام مداخله از طریق پوست (استنت گذاری)
**نحوه ی همکاری مشارکت کنندگان در این مطالعه**	بیماران با مشارکت در این طرح به صورت تصادفی به دو گروه دارودرمانی با تیکاگرول و دارودرمانی با ترکیب آسپیرین و کلوپیدوگرل بعد از استنت گذاری تقسیم میشوند. قبل از انجام طرح رضایت آگاهانه اخد میشود. نمونه گیری خون و بررسی مشخصات بیمار به طور کامل انجام میشود. بعد از انجام مداخله بیمار ماهیانه به صورت تلفنی مورد ارزیابی و پیگیری قرار میگیرد. هر دو ماه یکبار تا پایان شش ماه به صورت حضوری مورد ارزیابی و ویزیت پزشک قرار میگیرد.
**خونگیری**	یک مورد خونگیری قبل از انجام مداخله و یک مورد بعد از 6 ماه از انجام مداخله برای ارسال آزمایشات شامل آزمایش خون، عملکرد انعقادی، پروفایل چربی و آزمایشات الکترولیت انجام میگیرد.
**مزایای احتمالی**	کاهش خطرات خونریزی ناشی از مصرف آسپیرین می تواند از مزایای احتمالی این طرح باشد. شرکت بیماران در پژوهش می تواند به بهبود روش های تشخیصی و درمانی در آینده کمک کند.
**خطرات احتمالی**	افزایش خطرات گرفتگی رگ می تواند از خطرات احتمالی این طرح باشد.
**نحوه ی جبران خطرات**	با پایش مداوم و ویزیت اختصاصی بیماران در طی مطالعه شش ماهه، سعی میشود تا خطرات احتمالی به کمترین میزان ممکن برسد و در صورت احساس خطر مداخلات مورد نیاز به صورت آنی انجام گیرد.
**هزینه**	هزینه های این طرح تفاوتی با هزینه درمان روتین بیمار ندارد و هزینه های مداخلاتی توسط بیمار و بیمه تامین میگردد.
**روشهای جایگزین شرکت در این مطالعه**	در صورت عدم پذیرش طرح توسط بیمار، درمان روتین ترکیبی آسپیرین و کلوپیدوگرل برای بیمار پس از مداخله آغاز میشود.
**محرمانه بودن**	نتایج آزمایشات و اطلاعات طرح به صورت محرمانه نگهداری خواهد شد و تنها به صورت خصوصی به بیمار گزارش داده میشود. مجریان طرح پس از انجام مداخله نسبت به اطلاعات بیماران کور هستند و تنها از نوع داروی مصرفی بیمار بدون دانستن نام بیمار آگاه هستند. کمیته اخلاق دانشگاه علوم پزشکی شیراز با هدف نظارت بر رعایت حقوق شرکت کنندگان می تواند به اطلاعات ایشان دسترسی داشته باشد و همچنین گرفتن رضایت آگاهانه جهت استفاده از نمونه های اخذ شده در تحقیقات دیگر در آینده ضروریست.
**پاسخگویی به پرسشها**	بیمار میتواند تمام پرسش ها و پیگیری های خود را از طریق مطب مجری اول طرح (دکتر کجوری) واقع در شیراز، چمران، بلوار نیایش، ساختمان پزشکان با شماره ۰۷۱۳۶۵۴۰۰۶۸ انجام دهد.
**حق نپذیرفتن یا انصراف**	**شرکت من در مطالعه کاملاً اختیاری است و آزاد خواهم بود که از شرکت در مطالعه امتناع نموده یا هر زمان که مایل بودم بدون آنکه تغییری در نحوه رفتار پزشک درمانگر یا نحوه درمان و مراقبت از بیماری اینجانب ایجاد شود از پژوهش مذکور خارج شوم.**
**اطلاع رسانی، پیشنهادات**	**ضمن تشکر از همکاری شما در این پروژه پژوهشی لطفاً هرگونه نظرات، پیشنهادات و یا مشکلاتی در پروسه انجام این تحقیق وجود داشته است با شماره تلفن های 32122389 یا 32122438 دفتر کمیته اخلاق دانشگاه علوم پزشکی**

**و پیگیری مشکلات**	**شیراز و یا با ایمیل researchethic@sums.ac.ir و یا فکس به شماره 32122686 با ما در میان بگذارید. بـدیهی است پیگیری موارد مطرح شده بصورت کاملاً محرمانه توسط کمیته اخلاق در پژوهش های دانشگاه علـوم پزشـکی شیراز انجام می پذیرد.**

بیمار گرامی /شرکت کننده محترم : به اطلاع میرساند شما میتوانید با مراجعه به سایت مرکز ثبت کاآزمایی بالینی ایران به آدرس www.irct.ir از جزییات بیشتری در رابطه با این مطالعه آگاهی یابید .

شایان ذکر است کد ثبت این مطالعه در سایت پیشگفت به شرح زیر است .

کد ثبت مطالعه در مرکز ثبت کار آزمایی بالینی ایران :

((رضایت))

اینجانب .... با آگاهی کامل از موارد فوق رضایت می دهم که به عنوان یک فرد مورد مطالعه در این پژوهش شرکت نمایم.

کلیه اطلاعاتی که از من گرفته می شود و نیز نام من محرمانه باقی خواهد ماند و نتایج تحقیقات به صورت کلی و در قالب اطلاعات گروه مـورد مطالعـه منتشر می گردد و نتایج فردی در صورت نیاز بدون ذکر نام و مشخصات فردی عرضه خواهد گردید.

این موافقت مانع از اقدامات قانونی اینجانب درمقابل (نام واحد ذکر گردد) درصورتی که عملی خلاف و غیرانسانی انجام شود نخواهد بود.

امضاء و اثرانگشت **فرد مورد پژوهش** نام و نام خانوادگی، شماره تماس و امضاء **شاهد** نام و نام خانوادگی و امضاء **پژوهشگر**

(و یا قیم قانونی)

شماره تماس ثابت:

شماره تلفن همراه:

تاریخ:

توضیحات:

1- این فرم باید در سه نسخه تهیه شود، نسخه اول در محلی مطمئن بایگانی شود تا دستیابی به آن برای کنترل پایشگران یا کمیته اخلاق در پژوهش های علوم پزشکی آسان شـود، نسـخه دوم آن در اختیـار شـرکت کننده در طرح قرار گیرد و نسخه سوم در پرونده بالینی ضمیمه گردد.

2- اثرانگشت سبابه دست راست و درصورت عدم امکان اخذ آن، زیر اثر انگشت اخذ شده توضیح داده شود.

3- سن قانونی بالای 18 سال تمام می باشد.

4- گروه های آسیب پذیر شامل کودکان و نوزادان، زنان باردار و جنین، ناتوانان ذهنی، زنـدانیان و بیمـاران اورژانس افرادی هستند که نیاز به اخذ رضایت از قیم و سرپرست دارند.
