# Supplementary material for: Rationale and design of randomized non-inferiority clinical trial to compare the safety and efficacy of ticagrelor monotherapy with dual antiplatelet therapy in chronic coronary syndrome patients post percutaneous coronary intervention (TICALONE-TAHA10 Protocol)
Source: PLoS One. 2025 Jul 16;20(7):e0325663. doi: 10.1371/journal.pone.0325663 (PMC12266445; doi:10.1371/journal.pone.0325663)
Supplement: S1 Data — Appendix 1 - Baseline Characteristics Appendix 2 - Follow-up Variables Appendix 3 - Informed Consent Form Ethics Approval Funding Contract SPRITI checklist. (ZIP) [file pone.0325663.s001.zip › supporting data/ethics-English[1].pdf]

Shiraz University of Medical Sciences

School of Medicine - Shiraz University of Medical Sciences

Research Ethics Committees Certificate

Approval ID:	IR.SUMS.MED.REC.1403.150	Approval Date:	2024-06-01
Evaluated by:	Research Ethics Committees of School of Medicine - Shiraz University of Medical Sciences		
Status:	Approved		
Approval Statement:	The project was found to be in accordance to the ethical principles and the national norms and standards for conducting Medical Research in Iran.<br>Notice:<br>1. Although the proposal has been approved by the Biomedical Research Ethics Committee, meeting the professional and legal requirements is the sole responsibility of the PI and other project collaborators.<br>2. This certificate is reliant on the proposal/documents received by this committee on 2024-06-01. The committee must be notified by the PI as soon as the proposal/documents are modified.<br>3. Other Comments:<br>◦ It is required to register this research project in the Iranian Registry of Clinical Trial website with the following address: www.irct.ir		
Thesis Title:	A Single-center, Open-label, Randomized, 6-month, Non-inferiority Study to Compare the Safety and Efficacy of Ticagrelor monotherapy versus Dual Antiplatelet Therapy in Chronic Coronary Syndrome Patients Post PCI		
Supervisor:	Name: Javad Kojuri<br>Email: kojurij@yahoo.com		
Student:	Name: Davar Al Davod Zavalid<br>Email: davar_20z@yahoo.com		

Dr. Ramin Shiraly
Committee Director
School of Medicine - Shiraz University of Medical Sciences

Laleh Dehghanpisheh

Dr. Laleh Dehghanpisheh
Committee Secretary
School of Medicine - Shiraz University of Medical Sciences
