# Supplementary material for: Rationale and design of randomized non-inferiority clinical trial to compare the safety and efficacy of ticagrelor monotherapy with dual antiplatelet therapy in chronic coronary syndrome patients post percutaneous coronary intervention (TICALONE-TAHA10 Protocol)
Source: PLoS One. 2025 Jul 16;20(7):e0325663. doi: 10.1371/journal.pone.0325663 (PMC12266445; doi:10.1371/journal.pone.0325663)
Supplement: S1 Data — Appendix 1 - Baseline Characteristics Appendix 2 - Follow-up Variables Appendix 3 - Informed Consent Form Ethics Approval Funding Contract SPRITI checklist. (ZIP) [file pone.0325663.s001.zip › supporting data/funding-english[1].pdf]

IN THE NAME OF GOD
OFFICIAL TRANSLATION FROM PERSIAN
The IR Iran
Shiraz University of Medical Sciences
Vice-Chancellor for Research & Technology

CONTRACT FOR SHIRAZ UNIVERSITY OF MEDICAL SCIENCES RESEARCH PROJECTS

Project No.: 29781
Ethics Committee Approval Code: IR.SUMS.MED.REC.1403.150

Article 1) Parties to the Contract
This contract was made by and between Dr. JAVAD KOJURI as the executive manager and representative of executors of the research project whose names are written in the proposal (attachment 1) who is referred to hereinafter as the "researcher", domiciled at School of Medicine, Shiraz university of Medical Sciences, Tel. +989171115083, on one side and vice-chancellor for research and technology in Shiraz University of Medical Sciences, domiciled at Zand St., Shiraz, Iran, referred to hereinafter as the "vice-chancellor for research & technology" on the other side. Parties are obliged to follow the content as they sign the contract. The researcher cannot have the excuse of being unaware of the content.
Article 2) Object of Contract
Carrying out the research project titled as "Comparing the Safety and Affectivity of Ticagrelor Monotherapy with Standard Dual Antiplatelet Therapy in Patients with Chronic Coronary Syndrome after PCI: a Six-Month Monocentral Random Clinical Trial" according to the attached draft parts of which have been completed and signed by the executor and collaborators of the project and approved by the research council of the university on Jun. 10, 2024 with No. 29781.
Article 3) Researcher's Scope of Services
Scope of services and schedule must comply with the time-table in the draft. The researcher undertakes compensate for the losses in case there were any. If there were any delay in completing the object of contract, the vice-chancellor for research deduct up to 2% of the contract price for every day of delay in delivering the final report from the researcher's salary.
Note 1: The researcher allows the vice-chancellor for research to deduct the mentioned sum from his salary under supervision of the vice-chancellor for logistics.
Article 4) Period of Contract:
15 months from Jun. 10, 2024 to Sept. 12, 2025
Article 5) Price of Contract:
Total sum of IRR 118,486,800 which will be paid to the researcher according to article 6 after the costs of supervision for good and performance and other legal deductions are made.
Note 1: Supervision cost is IRR 0 which is included in the legal deductions.
Article 6) Payment Method
6-1- IRR 59,243,000 as the first payment after the draft is approved and the contract is signed
6-2- IRR 0 as the second payment after the first progress report is presented and approved by the supervisor
6-3- IRR 35,545,800 as the third payment after the final report is presented and approved by the supervisor
6-4- IRR 23,697,200 as the fourth payment after the article is published in a valid journal (or approved by authorities in charge) and after approval of the supervisor the net payment including the researcher's for IRR 23,697,200 and the supervisor's payment for IRR 0 will be made.

MORTEZA MASOUMNEJAD
مرتضی معصوم نژاد
مترجم رسمی زبان انگلیسی
قوه قضائیه - شیراز
شماره پروانه: ۱۰۴۴
CERTIFIED TRANSLATOR TO THE JUDICIARY, SHIRAZ, IRAN LICENSE NO. 1044

آدرس: میدان دانشجو، نبش خیابان ساحلی، ساختمان ارم
Address: Eram Bldg., Daneshjoo Sq., Shiraz, Iran
Tel:(+98) 071 071-32299094 Mobile:(+98) 9306684360 Email: 1044cto@gmail.com

Article 7) Using research Results

The vice-chancellor for research and technology is the intellectual proprietor of this project. Scientific outcome and financial benefits of the project will be determined upon mutual agreement.

Article 8) Other Expenses:

The researcher must pay all expenses of personnel, services, administrative, scientific and practical affairs and the vice-chancellor has no liability to pay so except for those mentioned in article 5. The researcher will be liable against claims made by the personnel.

Article 9) Obligations of the Researcher

9-1- Performing scientific activities related to the object of contract and accepting quantitative and qualitative responsibilities and carrying out the research on due time

9-2- Keeping and protecting the property and documents provided for him by the vice-chancellor and making the best use of them to complete the object of contract

9-3- Researcher must not present the documents and information he is provided with to other real or legal entities unless having the written agreement of the vice-chancellor. Otherwise, the vice-chancellor may take legal actions against him to preserve their rights.

9-4- The researcher is obliged to provide the vice-chancellor with the published article or the results of using the products of the project within one year. Otherwise, the vice-chancellor for research can make due decisions for the assigning the next projects to the researcher.

9-6- Providing the supervisors with required actions and acceptable facilities to do their supervision tasks

9-7- The researcher must carry out the project in person and cannot transfer all or part of the contract to a third party

9-8- In case of purchasing any non-consumable item (property), the researcher must provide the vice-chancellor for research with the identification plate number.

9-9- The researcher is liable against legal and administrative authorities for any incident related to the object of contract.

9-10- The researcher undertakes to transfer his authorities to another person in case his employment is cancelled or he is not able to fulfill the obligations as parties agree or to return the paid sums to the vice-chancellor for research and technology.

9-11- The executor undertakes not to publish the result in interviews or notifications before having the confirmation of the vice-chancellor for research and technology in clinical trial studies.

9-12- In medicinal studies, it is only possible to study the effects of herbal products or therapy methods after approval of vice-chancellor for research or having the required permissions..

Article 10) Obligations of Vice-chancellor for Research

10-1- Helping the researcher access articles or scientific texts which are not available in the country

10-2- Paying the total price of contract as mentioned in article 5

Article 11) Dispute Settlement

In case of any disputes in interpreting parts or all of the content of the contract, 3 professors of the university whom parties mutually agree upon will make the obligatory decision. In case the dispute remains unsettled, the parties can refer to legal authorities.

Article 12) Amendment, Supplement, Complement or Modification

Any change or modification in the contract and attachments or any supplementary or complementary contract can be made upon mutual agreement.

MORTEZA MASOUMNEJAD
مرتضی معصوم نژاد
مترجم رسمی زبان انگلیسی
قوه قضائیه ـ شیراز
شماره پروانه ۱۰۴۴
CERTIFIED TRANSLATOR TO THE JUDICIARY, SHIRAZ, IRAN LICENSE NO. 1044

جمهوری اسلامی ایران
قوهٔ قضائیه - اداره مترجمین رسمی

مرتضی معصوم نژاد
Official English Translator to the Judiciary
License No. 1044
Morteza Masoumnejad
To verify the authenticity of the translation, please scan the QR Code

Article 13) Contract Nullification:
The contract cannot be nullified by one party. Nullification may only be done according to regulations and in the case mentioned in 6-3.
Article 14) Attachments
This contract has one attachment (proposal) which is an inseparable part of the contract and must be signed by parties when the contract is signed.
Article 15) Parties' Address:
Parties' address is that written in article 1 and any change must be notified to the other party.
Article 16) Laws Superseding this Contract
This contract follows the laws of the IR Iran
Article 17) Special Conditions
In case of force majeure or any conditions which the parties are not able to control and which make completion of the project impossible, the period of contract can be extended upon mutual agreement. If in such cases the contract was nullified parties cannot claim for compensation of losses and clearance will be made upon mutual agreement.

This contract which includes 17 articles and 3 notes and 1 attachment was made in 3 copies with the same validity in Persian language on Jun. 19, 2024.
This contract was made according to Article 40 of the bylaw for employing faculty members and was signed by parties who are obliged to obey its content.

Researcher: Javad Kojuri (Signed)
Vice-Chancellor for Research & Technology: Mohammad Hashem Hashempur (Signed & Sealed)

True Translation Certified
Morteza Masoumnejad, Certified Translator
Jul. 31, 2024

MORTEZA MASOUMNEJAD
CERTIFIED TRANSLATOR TO THE JUDICIARY, SHIRAZ, IRAN LICENSE NO. 1044
مرتضی معصوم نژاد
مترجم رسمی زبان انگلیسی
قوه قضائیه - شیراز
شماره پروانه ۱۰۴۴

آدرس: میدان دانشجو، نبش خیابان ساحلی، ساختمان ارم
Address: Eram Bldg., Daneshjoo Sq., Shiraz, Iran
Email: 1044cto@gmail.com Mobi[illegible] +98) 9306684360 Tel:(+98) 071 071-32299094

جمهوری اسلامی ایران

« معاونت تحقیقات و فناوری »

قرارداد طرحهای پژوهشی دانشگاه علوم پزشکی شیراز

شماره طرح:۲۹۷۸۱

کد مصوب کمیته اخلاق: IR.SUMS.MED.REC.1403.150

ماده ۱: طرفین قرارداد

این قرارداد بین آقا/خانم دکتر **جواد کجوری** بعنوان مدیر اجرایی به نمایندگی از طرف مجریان طرح تحقیقاتی که در پیش نویس (Proposal) (پیوست شماره یک قرارداد) اسامی ایشان قید گردیده و از این پس پژوهشگر نامیده می شود به نشانی: **علوم پزشکی شیراز/دانشکده پزشکی/قلب و عروق** شماره تلفن/ همراه **۰۹۱۷۱۱۱۵۰۸۳** و از طرف دیگر معاونت تحقیقات و فناوری دانشگاه علوم پزشکی شیراز به نشانی: شیراز- خیابان زند- ساختمان مرکزی دانشگاه علوم پزشکی شیراز- طبقه هفتم که از این پس معاونت تحقیقات و فناوری نامیده می شود، منعقد می گردد و طرفین با امضاء این قرارداد خود را ملزم و متعهد به رعایت اجرای کامل و تمام مفاد آن می دانند و پژوهشگر در اجرای مفاد پیمان به عذر عدم اطلاع نمی تواند متعذر گردد.

ماده ۲: موضوع قرارداد

اجرای طرح پژوهشی با عنوان « **مقایسه ایمنی و اثربخشی تک درمانی تیکاگرلور در مقایسه با درمان ضد پلاکتی دوگانه استاندارد در بیماران مبتلا به سندرم کرونر مزمن پس از PCI؛ یک کار آزمایی بالینی تصادفی تک مرکزی شش ماهه** » مطابق با پیش نویس طرح پیوست که قسمتهای مختلف آن توسط مجری طرح تکمیل و به امضاء رسیده و بر اساس مقررات مربوط توسط شورای پژوهشی دانشگاه در تاریخ **۱۴۰۳/۰۳/۲۱** به تصویب رسیده و با شماره **۲۹۷۸۱** ثبت شده است.

ماده ۳: حدود و خدمات پژوهشگر

حدود خدمات پژوهشگر و مراحل انجام کار مطابق با جدول زمان بندی مندرج در پیش نویس طرح و در ۲ مرحله گزارش می باشد. پژوهشگر متعهد می گردد چنانچه موجب ضرر و زیانی گرددنسبت به جبران آن اقدام نماید و در صورتی که تاخیری در تحویل موضوع قرارداد پیش آید معاونت پژوهشی به صلاحدید خود مختار خواهد بود تا سقف ۰/۲ درصد از مبلغ کل قرارداد به ازاء هر روز تاخیر در ارسال گزارش نهایی (مطابق جدول گانت) جهت جبران خسارت از محل حقوق و مزایای پژوهشگر ضمن مطلع نمودن وی و هماهنگی با واحدهای ذیربط دانشگاه اخذ نماید.

تبصره ۱: پژوهشگر به معاونت پژوهشی اجازه می دهد نسبت به مطالبات مربوط به ضرر وزیان ناشی از عدم اجرای طرح از محل حقوق و مزایای وی با هماهنگی با معاونت پشتیبانی اقدام نماید.

ماده ۴: مدت انجام قرارداد

مدت انجام این قرارداد **۱۵** ماه بوده و از تاریخ **۱۴۰۳/۰۳/۲۱** شروع و در تاریخ **۱۴۰۴/۰۶/۲۱** خاتمه می پذیرد.

ماده ۵: مبلغ قرارداد

مبلغ کل این قرارداد **۱۱۸،۴۸۶،۸۰۰ ریال** (**یکصد و هجده میلیون و چهارصد و هشتاد و شش هزار و هشتصد ریال**) است که **۱۱۸،۴۸۶،۰۰۰ ریال یکصد و هجده میلیون و چهارصد و هشتاد و شش هزار ریال**) آن سهم معاونت تحقیقات و فناوری و **۰ ریال** مجموع اعتبار جذب شده از محل گرنت یا سایر سازمانها می باشد و پس از کسر هزینه نظارت بر حسن اجرای طرح (حق نظارت بر اساس توافق معاونت با ناظر یا ناظرین تعیین و پرداخت می گردد) و مابقی کسورات قانونی (که بموجب قوانین و مقررات موجود و یا آنچه که بعداً وضع خواهد شد و به این قرارداد تعلق می گیرد) به شرح ماده ۶ و تبصره های ذیل ماده ۵ به پژوهشگر پرداخت می شود.

تبصره ۱: مبلغ ۰ ریال به عنوان هزینه نظارت در نظر گرفته شده است. کسورات قانونی شامل هزینه نظارت می باشد.

MORTEZA MASOUMNEJAD
مرتضی معصوم نژاد
مترجم رسمی زبان انگلیسی
قوه قضاییه - شیراز
شماره پروانه ۱۰۴۴
CERTIFIED TRANSLATOR TO THE JUDICIARY, SHIRAZ, IRAN LICENSE NO.1044

رونوشت با اصل برابر است
True Copy Certified

ماده ۶: مراحل پرداخت

۶-۱: مبلغ **۵۹،۲۴۳،۰۰۰ ریال** بعنوان قسط مرحله یک که پس از تأیید پیش نویس طرح و امضاء قرارداد پرداخت می گردد.

۶-۲: مبلغ **۰ ریال** بعنوان قسط مرحله دو پس از ارائه گزارش پیشرفت اول و تأیید ناظر طرح پرداخت می گردد.

۶-۳: مبلغ **۳۵،۵۴۵،۸۰۰ ریال** بعنوان قسط مرحله سه پس از ارائه گزارش نهایی و تأیید ناظر طرح پرداخت می گردد.

۶-۴: مبلغ **۲۳،۶۹۷،۲۰۰ ریال** بعنوان مرحله چهار پس از ارائه مقاله چاپ شده در مجلات معتبر علمی (و یا تأییدیه مراجع ذیصلاح در خصوص طرح های مداخله ای ، ثبت ایده، اختراع معتبر، ارائه خلاصه مقاله در کنگره مربوط به طرحهای کمیته تحقیقات دانشجویی) و پس از تأیید نهایی ناظر طرح مبالغ خالص شامل سهم پژوهشگر به میزان **۲۳،۶۹۷،۲۰۰ ریال** و حق الزحمه ناظر به میزان ۰ **ریال** پرداخت می گردد.

ماده ۷: استفاده از نتایج طرح

مالکیت معنوی داده ها و خروجی پروژه موضوع این قرارداد به معاونت تحقیقات و فناوری دانشگاه علوم پزشکی شیراز تعلق دارد و بروندادهای علمی و منافع مادی ناشی از طرح و بهره برداری از آن ها بر اساس توافق بین پژوهشگر و معاونت تعیین خواهد شد.

ماده ۸: سایر هزینه ها

پژوهشگر کلیه هزینه های پرسنلی، خدماتی، اداری، علمی و عملی و غیره را پرداخت می نماید و معاونت هیچگونه تعهدی بجز آنچه در ماده ۵ آمده نخواهد داشت. پژوهشگر در قبال دعاوی پرسنل و اشخاص مطروحه در مراجع قضایی و اداره کار و امور اجتماعی جوابگو خواهد بود.

ماده ۹ : تعهدات پژوهشگر

۹-۱: انجام فعالیتهای علمی و تحقیقات کافی در چارچوب اصول علمی و اخلاقی مدون مرتبط با موضوع این قرارداد توسط پژوهشگر و پذیرش مسئولیت کمی و کیفی و انجام به موقع پژوهش.

۹-۲: رعایت امانت و حفظ اموال و مدارکی که معاونت در اختیار پژوهشگر قرار می دهد و استفاده بهینه از آن برای انجام موضوع قرارداد.

۹-۳: عدم ارائه اسناد و مدارک و اطلاعاتی که به مناسبت انجام پژوهش کسب می نماید به اشخاص حقیقی یا حقوقی غیر، مگر با کسب اجازه کتبی از معاونت، در غیر اینصورت معاونت جهت استیفای حقوق خود اقدام قانونی علیه پژوهشگر معمول خواهد داشت.

۹-۴:پژوهشگر موظف است ظرف مدت یک سال پس از ارائه گزارش نهایی، مدرک اعلام پذیرش یا اصل مقاله چاپ شده و یا نتیجه بررسی یا بکارگیری محصول طرح تحقیقاتی را از مراجع ذینفع استعلام نموده و به معاونت ارائه نماید در غیر اینصورت مدیریت امور پژوهشی دانشگاه در خصوص اجرای طرحهای مصوب بعدی نامبرده بعنوان طرح دهنده و یا مجری مختار به اخذ تصمیم است.

۶-۹: ایجاد امکانات لازم و تسهیلات قابل قبول جهت نظارت علمی توسط نمایندگان معاونت و ناظرین طرح.

[illegible] شخصا عهده دار انجام کار موضوع این قرارداد است و حق واگذاری به غیر را ندارد.

[illegible] موظف است در صورت خرید وسایل غیر مصرفی (اموالی) مندرج در پیش نویس طرح شماره پلاک اموال مذکور را به معاونت پژوهشی ارائه نماید.

[illegible] درصورت بروز هرگونه حادثه یا اتفاق مرتبط با اجرای طرح، مسئولیت پاسخگویی نزد کلیه مراجع اعم از قضائی، اداری و ... را به عهده می گیرد و کلیه خسارات مادی و غیر مادی [illegible] را اعم از حقیقی و حقوقی جبران می نماید.

[illegible] مصوبه جلسه ۳۶ هیئت رئیسه دانشگاه مورخ ۱۴۰۰/۱۱/۲۵، پژوهشگر متعهد می گردد در صورت قطع رابطه استخدام یا عدم امکان اجرای تعهدات این قرارداد، اختیار اجرای [illegible] دیگری بر اساس توافق طرفین و با موافقت معاونت تحقیقات و فناوری دانشگاه واگذار کند یا مبالغ دریافتی را به حساب درآمد اختصاصی معاونت تحقیقات و فناوری دانشگاه مسترد نماید.

۱۱-۹: در مطالعات کارآزمایی بالینی و مداخله ای مجری متعهد می گردد از هرگونه انتشار نتایج د ر قالب مصاحبه ها ی خبری، اعلام عمومی و رسانه ای قبل از تایید معاونت تحقیقات و فناوری خودداری نماید.

۱۲-۹: در مطالعات دارویی، بررسی اثر بخشی فرآورده های گیاهی و یا روش های درمانی تنها پس از تایید معاونت تحقیقات و فناوری دانشگاه و کسب مجوزهای لازم مندرج در قوانین بالا دستی، اعلام عمومی و انتشار میسر می باشد.

ماده ۱۰: تعهدات معاونت

۱-۱۰: در حد امکان و در ارتباط با موضوع قرارداد کمک به تهیه مقالات و سایر متون علمی که در کشور غیر قابل دسترسی باشد

۲-۱۰: پرداخت کامل مبلغ قرارداد بر اساس مراحل مندرج در ماده ۵ پس از کسر کسورات متعلقه.

ماده ۱۱ : حل اختلاف

در مواردی که برای طرفین این قرارداد در تعبیر و تفسیر و یا اجرای کامل یا قسمتی از قرارداد اختلاف نظری حادث گردد، موضوع توسط ۳ نفر از اساتید مرضی الطرفین دانشگاه بررسی و رای آنها برای طرفین لازم الاجرا است و در صورتیکه مساعی طرفین و نمایندگان آنان به نتیجه نرسد، هر یک از طرفین حق خواهد داشت جهت احقاق حق خود به مراجع ذیصلاح قضایی مراجعه نماید.

ماده ۱۲ : اصلاحیه، متمم، مکمل یا تغییر

هر گونه اصلاح یا تغییر در مواردی از این قرارداد و پیوست های مربوطه و یا نیاز به الحاق هر گونه متمم یا مکمل به قرارداد و پیوستها با جلب توافق متعاقدین میسر خواهد بود.

ماده ۱۳ : فسخ قرارداد

فسخ یک جانبه قرارداد از سوی هر یک از طرفین قابل پذیرش نبوده و فسخ قرارداد به غیر از موارد قانونی، فقط با توافق طرفین امکان پذیر است.

ماده ۱۴ : ضمائم قرارداد

این قرارداد شامل یک پیوست (پروپوزال پیوست) بعنوان شرح خدمات این قرارداد که جزء لاینفک قرارداد محسوب شده و در زمان امضای قرارداد پیوست ها نیز باید به امضاء متعاقدین رسیده باشد.

ماده ۱۵ : اقامتگاه طرفین

اقامتگاه قانونی طرفین همان نشانی مندرج در ماده یک قرارداد بوده و در موارد لزوم و به منظور ابلاغ مکاتبات، مورد استناد خواهد بود و در صورتیکه در طول قرارداد نشانی متعاقدین تغییر یابد طرفین باید رسماً و کتباً مراتب را به یکدیگر ابلاغ نمایند و تا قبل از ابلاغ رسمی نشانی قبلی معتبر خواهد بود.

ماده ۱۶ : قانون حاکم بر قرارداد

این قرارداد از هر نظر تابع قوانین جمهوری اسلامی ایران می باشد.

ماده ۱۷ : شرایط خاص

در مواردی که به علل ناشی از شرایط قهری (شرایط خاص) که رفع آن خارج از حیطه توانایی و اقتدار طرفین قرارداد باشد و انجام بخشی از قرارداد یا تمام آن غیر ممکن گردد، در صورت موافقت طرفین به مدت زمان اجرای قرارداد اضافه خواهد شد و در صورت فسخ قرارداد هیچیک از طرفین حق مطالبه خسارت وارده را نخواهد داشت و اقدامات پس از فسخ از قبیل تسویه حساب با توافق طرفین صورت می گیرد.

این قرارداد در ۱۷ ماده و ۳ تبصره و یک پیوست به شرح ماده ۱۴ و به زبان فارسی و در سه نسخه در تاریخ ۱۴۰۳/۰۳/۳۰ تنظیم گردید و کلیه نسخه های آن دارای اعتبار یکسان و برابر می باشد.

این قرارداد با استناد به ماده ۴۰ آئین نامه استخدامی هیئت علمی دانشگاه و موسسات آموزش عالی و پژوهشی کشور و تبصره های ذیل آن به امضای طرفین رسیده و متعاقدین خود را ملزم و متعهد به اجرای کلیه مفاد آن و پیوست های مربوطه می دانند.

○ **اینجانب ....**

الف) اعلام می دارم در خصوص الزام ثبت آینده نگر این مطالعه در سامانه مرکز ثبت کارآزمایی های بالینی ایران (IRCT) حداکثر تا یک ماه پس از تاریخ تصویب طرح به من اطلاع رسانی شده است و متعهد می گردم تا زمان تعلق شماره ثبت مطالعه از آن مرکز اقدام به شروع مطالعه ننمایم و هرگونه عواقب حقوقی و اخلاقی تاخیر در ثبت مطالعه و یا شروع مطالعه پیش از تعلق کد به عهده من می باشد و معاونت تحقیقات و فناوری دانشگاه مسوولیتی در این مورد ندارد.

ب)متعهد می شوم کلیه فرمهای رضایت آگاهانه اخذ شده را مطابق با جدول زمانبندی ارایه شده در طرح پژوهشی و بلافاصله پس از پایان بیمارگیری در قالب یک فایل در قسمت گزارش پیشرفت طرح بارگذاری کنم و درصورتی که به هر دلیلی مدت زمان بیمارگیری بیش از زمان پیش بینی شده در پروپوزال به طول انجامید، طی مکاتبه با معاون تحقیقات و فناوری مراتب را با ذکر دلیل اطلاع رسانی نمایم.

نام و نام خانوادگی مجری طرح:
دکتر جواد کجوری
مهر و امضای پژوهشگر

دکتر محمدهاشم هاشم پور
معاون تحقیقات و فناوری دانشگاه علوم پزشکی شیراز

دانشگاه علوم پزشکی و خدمات بهداشتی درمانی شیراز
معاون
تحقیقات و فناوری دانشگاه

MORTEZA MASOUMNEJAD
مرتضی معصوم نژاد
مترجم رسمی زبان انگلیسی
قوه قضائیه - شیراز
شماره پروانه ۱۰۴۴
CERTIFIED TRANSLATOR TO THE JUDICIARY, SHIRAZ, IRAN LICENSE NO.1044

- نسخه اول- مدیر اجرایی طرح
- نسخه دوم - مدیریت توسعه و ارزیابی تحقیقات جهت درج در پرونده
- نسخه سوم- حسابداری معاونت تحقیقات و فناوری جهت اقدام
- تصویر قرارداد- دبیرخانه حوزه معاونت تحقیقات و فناوری

رونوشت با اصل برابر است
True Copy Certified
