# Supplementary material for: Rationale and design of randomized non-inferiority clinical trial to compare the safety and efficacy of ticagrelor monotherapy with dual antiplatelet therapy in chronic coronary syndrome patients post percutaneous coronary intervention (TICALONE-TAHA10 Protocol)
Source: PLoS One. 2025 Jul 16;20(7):e0325663. doi: 10.1371/journal.pone.0325663 (PMC12266445; doi:10.1371/journal.pone.0325663)
Supplement: S1 Data — Appendix 1 - Baseline Characteristics Appendix 2 - Follow-up Variables Appendix 3 - Informed Consent Form Ethics Approval Funding Contract SPRITI checklist. (ZIP) [file pone.0325663.s001.zip › supporting data/Study_protocol[1].pdf]

Study protocol

Title: A Single-center, double blinded, Randomized, Non-inferiority Study to Compare the Safety and Efficacy of Ticagrelor monotherapy versus Dual Antiplatelet Therapy in Chronic Coronary Syndrome Patients Post PCI

Tracking Code: 29781

Principal Investigator: Javad Kojuri

Specialty: Cardiology

First Author (Student): Davar Aldavood

Student ID: 9312657009

Ethics Code: IR.SUMS.MED.REC.1403.150

Project Type: Interventional - Randomized Controlled Trial (RCT)

Initial Registration Date: 2023/12/26 (Persian Date: 1402/10/05)

Submission Date: 2024/01/04 (Persian Date: 1402/10/15)

Last Revision Date: 2024/05/29 (Persian Date: 1403/03/08)

Primary Receiving Center: Shiraz University of Medical Sciences / Advanced Medical Faculty Subspecialty Office / Cardiology

General Project Information:

Persian Title:

مقایسه ایمنی و اثربخشی تک درمانی تیکاگرلور در مقایسه با درمان ضد پلاکتی دوگانه استاندارد در بیماران مبتلا به سندرم کرونر مزمن پس از PCI؛ یک کار آزمایی بالینی تصادفی تک مرکزی

English Title:

A Single-center, double blinded, Randomized, Non-inferiority Study to Compare the Safety and Efficacy of Ticagrelor Monotherapy versus Dual Antiplatelet Therapy in Chronic Coronary Syndrome Patients Post PCI

Project Type:

Applied

Thesis Level:

Postdoctoral Fellowship (Subspecialty)

Type of Study:

Interventional - Randomized Controlled Trial (RCT)

Student Status:

Self-funded

Student ID Number:

40212143002

Year of Entry (4 digits):

1402 (2023-2024 in Gregorian calendar)

Abstract:

This comprehensive randomized, open-label, non-inferiority trial focuses on evaluating the comparative efficacy and safety of ticagrelor monotherapy versus traditional dual antiplatelet therapy (DAPT) in patients with chronic coronary syndrome (CCS) undergoing percutaneous coronary intervention (PCI). The primary objective is to assess whether ticagrelor alone can sufficiently balance the risks of ischemic events and bleeding, a crucial consideration in post-PCI management for CCS patients. The study will be conducted at a single center in Shiraz, Iran, and involves two patient cohorts: one receiving ticagrelor as sole antiplatelet therapy post-PCI and the other adhering to the standard DAPT regimen. The primary endpoint is a composite of major adverse cardiac events, with secondary endpoints including bleeding complications and overall patient mortality. This trial's outcomes are anticipated to provide pivotal insights into the optimal antiplatelet strategy for CCS patients post-PCI, potentially guiding future clinical practice and therapeutic guidelines.

Keywords:

Chronic Coronary Syndrome, Percutaneous Coronary Intervention, Ticagrelor Monotherapy, Dual Antiplatelet Therapy, Ischemic Events, Bleeding Risks, Cardiac Adverse Events, Non-Inferiority Trial, Cardiovascular Pharmacotherapy.

Registry Data Usage:

No

Cohort Data Usage:

No

International Collaboration:

No

Supervisor Information:

- **First Name:** Javad
- **Last Name:** Kojuri
- **Role:** Primary Supervisor
- **Affiliation:** Shiraz University of Medical Sciences / Faculty of Medicine / Cardiology

---

Collaborators and Students:

Collaborators and Students (Former Team Members):

1. **Name:** Davar
   Last Name: Aldavood
   Affiliation: Shiraz University of Medical Sciences / Faculty of Pharmacy / Other fields (specific to non-faculty students and collaborators)
   Role in Project: Thesis Writing Student
   Specialty: Cardiology
2. **Name:** Seyed Alireza
   Last Name: Mirhosseini
   Affiliation: Shiraz University of Medical Sciences / Faculty of Medicine / MPH (Master of Public Health)
   Role in Project: Second Investigator

3. **Name:** Mohammadreza
   Last Name: Akbari
   Affiliation: Shiraz University of Medical Sciences / Vice Chancellor for Research and Technology - Grants / Other fields (specific to non-faculty students and collaborators)
   Role in Project: Scientific Collaborator

Problem Statement

Background, Literature Review, and Need for the Study

Dual antiplatelet therapy (DAPT), consisting of aspirin and clopidogrel, has been a cornerstone in the management of acute and chronic coronary syndrome (CCS) patients following percutaneous coronary intervention (PCI) since 2000 (1, 2). This therapy aims to mitigate the risk of thrombosis, associated with peri-procedural myocardial damage and stent-related complications. Aspirin, in combination with P2Y12 inhibitors, plays a critical role in long-term ischemic prevention by influencing the progression and destabilization of atherosclerotic plaques (3).

Advancements in antiplatelet therapy have evolved from ticlopidine to clopidogrel and, subsequently, to more potent agents such as prasugrel and ticagrelor, offering superior efficacy and faster action onset (4-8). Studies like PLATO have demonstrated ticagrelor's enhanced efficacy and safety compared to traditional treatments for acute coronary syndrome (ACS) patients (9).

Selecting the appropriate post-PCI regimen requires balancing ischemic and bleeding risks (10). Procedural and technical factors also significantly influence these risks (11-13). Numerous trials have explored optimal post-PCI regimens to minimize ischemic risk. However, the advent of antithrombotic stents and the observation that ischemic risks peak immediately post-PCI and decrease over time—while bleeding risks remain constant—have shifted the focus towards reducing bleeding risks (3).

This shift has prompted trials such as GLOBAL-LEADERS, TWILIGHT, SMART-CHOICE, STOP-DAPT-2, and TICO, which investigate alternatives like P2Y12 monotherapy and other regimens. These trials report differences in major adverse cardiovascular events (MACE) and significant variations in bleeding risk (14-16).

A significant portion of participants in these trials were CCS patients who had undergone PCI (17). Revascularization decisions for CCS patients depend on symptom severity, established ischemia, stenosis diameter, infarction area, cardiac contractility (EF), and coronary blood flow rate (18).

Future research seeks to explore novel antiplatelet agents, alternative regimens, and aspirin-free therapies. Aspirin-free antiplatelet therapy is hypothesized to reduce bleeding risks without increasing thrombosis risk. This hypothesis needs thorough investigation to generate evidence for improving treatment protocols.

References:

1. Levine GN, Bates ER, Bittl JA, Brindis RG, Fihn SD, Fleisher LA, et al. 2016 ACC/AHA guideline focused update on duration of dual antiplatelet therapy in patients with coronary artery disease: A report of the American College of Cardiology/American Heart Association Task Force on Clinical Practice Guidelines. *Journal of Thoracic and Cardiovascular Surgery.* 2016;152(5):1243-75.

2. Valgimigli M, Bueno H, Byrne RA, Collet JP, Costa F, Jeppsson A, et al. 2017 ESC focused update on dual antiplatelet therapy in coronary artery disease developed in collaboration with EACTS: The Task Force for dual antiplatelet therapy in coronary artery disease of the European Society of Cardiology (ESC) and of the European Association for Cardio-Thoracic Surgery (EACTS). *European Heart Journal.* 2018;39(3):213-60.
3. Angiolillo DJ, Galli M, Collet JP, Kastrati A, O'Donoghue ML. Antiplatelet therapy after percutaneous coronary intervention. *EuroIntervention.* 2022;17(17)

.

4. Bertrand ME, Rupprecht HJ, Urban P, Gershlick AH, Investigators C. Double-blind study of the safety of clopidogrel with and without a loading dose in combination with aspirin compared with ticlopidine in combination with aspirin after coronary stenting: The Clopidogrel Aspirin Stent International Cooperative Study (CLASSICS). *Circulation.* 2000;102(6):624-9.
5. Brandt JT, Payne CD, Wiviott SD, Weerakkody G, Farid NA, Small DS, et al. A comparison of prasugrel and clopidogrel loading doses on platelet function: Magnitude of platelet inhibition is related to active metabolite formation. *American Heart Journal.* 2007;153(1):66.e9-16.
6. Jakubowski JA, Payne CD, Brandt JT, Weerakkody GJ, Farid NA, Small DS, et al. The platelet inhibitory effects and pharmacokinetics of prasugrel after administration of loading and maintenance doses in healthy subjects. *Journal of Cardiovascular Pharmacology.* 2006;47(3):377-84.
7. Wiviott SD, Trenk D, Frelinger AL, O'Donoghue M, Neumann FJ, Michelson AD, et al. Prasugrel compared with high loading- and maintenance-dose clopidogrel in patients with planned percutaneous coronary intervention: The Prasugrel in Comparison to Clopidogrel for Inhibition of Platelet Activation and Aggregation-Thrombolysis in Myocardial Infarction 44 trial. *Circulation.* 2007;116(25):2923-32.
8. Angiolillo DJ, Ueno M, Goto S. Basic principles of platelet biology and clinical implications. *Circulation Journal.* 2010;74(4):597-607.
9. Wallentin L, Becker RC, Budaj A, Cannon CP, Emanuelsson H, Held C, et al. Ticagrelor versus clopidogrel in patients with acute coronary syndromes. *New England Journal of Medicine.* 2009;361(11):1045-57.
10. Galli M, Gargiulo G. Towards a personalized selection of antithrombotic agents in patients undergoing PCI: The role of clinical presentation in tools for risk assessment. *Journal of Thrombosis and Thrombolysis.* 2022;53(2):495-8.
11. Capodanno D, Bhatt DL, Gibson CM, James S, Kimura T, Mehran R, et al. Bleeding avoidance strategies in percutaneous coronary intervention. *Nature Reviews Cardiology.* 2022;19(2):117-32.

12. Galli M, Andreotti F, D'Amario D, Vergallo R, Vescovo GM, Giraldi L, et al. Antithrombotic therapy in the early phase of non-ST-elevation acute coronary syndromes: A systematic review and meta-analysis. *European Heart Journal - Cardiovascular Pharmacotherapy.* 2020;6(1):43-56.

13. Giustino G, Chieffo A, Palmerini T, Valgimigli M, Feres F, Abizaid A, et al. Efficacy and safety of dual antiplatelet therapy after complex PCI. *Journal of the American College of Cardiology.* 2016;68(17):1851-64.

14. O'Donoghue ML, Murphy SA, Sabatine MS. The safety and efficacy of aspirin discontinuation on a background of a P2Y12 inhibitor in patients after percutaneous coronary intervention: A systematic review and meta-analysis. *Circulation.* 2020;142(6):538-45.

15. Capodanno D, Mehran R, Valgimigli M, Baber U, Windecker S, Vranckx P, et al. Aspirin-free strategies in cardiovascular disease and cardioembolic stroke prevention. *Nature Reviews Cardiology.* 2018;15(8):480-96.

16. Benenati S, Galli M, De Marzo V, Pescetelli F, Toma M, Andreotti F, et al. Very short vs. long dual antiplatelet therapy after second-generation drug-eluting stents in 35,785 patients undergoing percutaneous coronary interventions: A meta-analysis of randomized controlled trials. *European Heart Journal - Cardiovascular Pharmacotherapy.* 2021;7(2):86-93.

17. Tomaniak M, Storey RF, Serruys PW. Aspirin-free antiplatelet regimens after PCI: When is it best to stop aspirin and who could ultimately benefit? *EuroIntervention.* 2020;15(13):1125-9.

18. Knuuti J, Wijns W, Saraste A, Capodanno D, Barbato E, Funck-Brentano C, et al. 2019 ESC Guidelines for the diagnosis and management of chronic coronary syndromes. *European Heart Journal.* 2020;41(3):407-77.

Study Variables:

Variable Name	Role	Type	Scale	Scientific Definition	Measurement Method
**Cardiac Death Incidence**	Dependent	Quantitative / Discrete	Number	Death due to a cardiovascular cause. Presence or absence of cardiac death in both groups.	Follow-up sessions (direct inquiry).
**Myocardial Infarction (MI) Incidence**	Dependent	Quantitative / Discrete	Number	Occurrence of MI in both treatment groups.	Follow-up sessions (direct inquiry).
**Bleeding Risk**	Dependent	Quantitative / Discrete	Number	Incidence of bleeding events, categorized by severity.	Follow-up sessions (direct inquiry).
**All-Cause Death Incidence**	Dependent	Quantitative / Discrete	Number	Occurrence of deaths from any cause.	Follow-up sessions (direct inquiry).
**Stroke Incidence**	Dependent	Quantitative / Discrete	Number	Types of stroke (ischemic, hemorrhagic, unknown) occurrences.	Follow-up sessions (direct inquiry).
**Need for Re-revascularization**	Dependent	Quantitative / Discrete	Number	Frequency of patients requiring re-revascularization.	Follow-up sessions (direct inquiry).
**Incidence of Stent Thrombosis**	Dependent	Quantitative / Discrete	Number	Occurrence of stent thrombosis after PCI.	Follow-up sessions (direct inquiry).
**Treatment Type**	Independent	Qualitative / Nominal	Other	Ticagrelor monotherapy vs. conventional DAPT.	Randomization (allocation).
**Patient Age**	Independent	Quantitative / Continuous	Number	Age of patients at the time of treatment.	Direct inquiry.
**Gender**	Independent	Qualitative / Nominal	Other	Sex.	Direct inquiry.
**Previous Cardiovascular Diseases**	Independent	Qualitative / Nominal	Other	History of heart attacks, angina, heart failure, or	Patient medical history and documents.

				other cardiac conditions.	
**Hypertension**	Independent	Qualitative / Nominal	Other	Long-standing or poorly controlled high blood pressure.	Patient medical history and documents.
**Dyslipidemia**	Independent	Qualitative / Nominal	Other	Abnormal cholesterol levels.	Patient medical history and documents.
**Diabetes Mellitus**	Independent	Qualitative / Nominal	Other	Both Type 1 and Type 2 diabetes.	Patient medical history and documents.
**Stroke or Transient Ischemic Attacks (TIA)**	Independent	Qualitative / Nominal	Other	Previous incidents of stroke or mini-strokes.	Patient medical history and documents.
**Peripheral Artery Disease (PAD)**	Independent	Qualitative / Nominal	Other	Conditions affecting blood flow to limbs.	Patient medical history and documents.
**Chronic Kidney Disease**	Independent	Qualitative / Nominal	Other	Presence of kidney damage or an estimated glomerular filtration rate (eGFR) < 60 ml/min/1.73 m^2 for ≥ 3 months.	Patient medical history and documents.
**Arrhythmias**	Independent	Qualitative / Nominal	Other	History of irregular heartbeat.	Patient medical history and documents.
**Previous Interventions**	Independent	Qualitative / Nominal	Other	Any prior cardiac surgeries or interventions, like stenting.	Patient medical history and documents.
**CABG**	Independent	Qualitative / Nominal	Other	Coronary artery bypass graft surgery.	Patient medical history and documents.
**HF**	Independent	Qualitative / Nominal	Other	Ejection fraction below 40%.	Patient medical history and echocardiography.
**NYHA Class**	Independent	Qualitative / Ordinal	Other	Classification of the extent of heart failure.	Patient medical history and documents.

**Previous CAD**	Independent	Qualitative / Nominal	Other	Coronary artery disease impacting heart blood supply.	Patient medical history and documents.
**Smoking Status**	Independent	Qualitative / Nominal	Other	Smoking cigarettes.	Patient social history.
**Body Mass Index (BMI)**	Independent	Quantitative / Continuous	Number	A person's weight in kilograms divided by the square of height in meters.	Weight and height measurements.
**Drug History**	Independent	Qualitative / Nominal	Other	Drug consumption by the patient.	Patient history and documents.

Study Objectives

General Objective

To comprehensively evaluate the safety and efficacy of ticagrelor monotherapy compared to the conventional dual antiplatelet therapy (DAPT) regimen in chronic coronary syndrome (CCS) patients post-percutaneous coronary intervention (PCI).

Specific Objectives

1. To assess and compare the incidence of cardiac death in CCS patients treated with ticagrelor monotherapy versus conventional DAPT post-PCI.
2. To evaluate the occurrence of myocardial infarction (MI) in patients receiving ticagrelor monotherapy compared to those on DAPT.
3. To quantify and compare the bleeding risk associated with ticagrelor monotherapy versus DAPT in this patient cohort.
4. To determine and contrast the incidence of all-cause mortality in patients undergoing ticagrelor monotherapy with those on DAPT.
5. To compare the incidence of different types of strokes (ischemic, hemorrhagic, and unknown) in CCS patients treated with ticagrelor monotherapy and DAPT.
6. To examine the need for re-revascularization in patients treated with ticagrelor monotherapy versus DAPT.
7. To analyze the incidence of cardiac death among CCS patients on conventional DAPT post-PCI, comparing it with ticagrelor monotherapy outcomes.

Practical Objectives

This study posits that ticagrelor monotherapy will demonstrate non-inferiority compared to conventional DAPT in balancing the reduction of major adverse cardiovascular events and bleeding risks in CCS patients post-PCI.

Hypotheses and Research Questions

Hypothesis

- Ticagrelor monotherapy is non-inferior to conventional DAPT in terms of safety and efficacy for reducing adverse cardiovascular events and bleeding risks in CCS patients after PCI.

Research Questions

1. Is there a difference in the incidence of cardiac death in CCS patients treated with ticagrelor monotherapy versus conventional DAPT post-PCI?
2. Is there a difference in the occurrence of myocardial infarction (MI) in patients receiving ticagrelor monotherapy compared to those on DAPT?
3. Is there a difference in the bleeding risk associated with ticagrelor monotherapy versus DAPT in this patient cohort?
4. Is there a difference in the incidence of all-cause mortality in patients undergoing ticagrelor monotherapy compared with those on DAPT?
5. Is there a difference in the incidence of different types of strokes (ischemic, hemorrhagic, and unknown) in CCS patients treated with ticagrelor monotherapy and DAPT?
6. Is there a difference in the need for re-revascularization in patients treated with ticagrelor monotherapy versus DAPT?
7. Is there a difference in the incidence of cardiac death among CCS patients on conventional DAPT post-PCI, comparing it with ticagrelor monotherapy outcomes?

Study Type

- Interventional - Randomized Controlled Trial (RCT)

Sample Size

- **Case Group:** 2700 participants
- **Control Group:** 2700 participants
- **Total Sample Size:** 5400 participants

Study Design

- A randomized, two-arm, double blinded, non-inferiority trial with a 1:1 allocation between ticagrelor monotherapy and conventional dual antiplatelet therapy (DAPT).
- Conducted at a single center in Shiraz, Iran.

Eligibility Criteria

Inclusion Criteria:

1. Male or female, above 20 years of age, undergoing PCI with drug-eluting stent (DES) for chronic coronary syndrome (CCS).
2. Patients providing written informed consent approved by the ethics committee.

Exclusion Criteria:

1. Contraindication to aspirin, clopidogrel, ticagrelor, or related drugs.
2. Atrial fibrillation or oral anticoagulant therapy.
3. Concurrent use of strong CYP3A inhibitors, inducers, or substrates with narrow therapeutic indices.
4. Females of childbearing potential without negative pregnancy test or unwilling to use contraception.
5. Unsuccessful PCI or suboptimal stent placement.
6. Acute coronary syndrome within the last 12 months.
7. History of stent thrombosis or stroke.
8. Conditions like severe anemia, active bleeding, intracranial hemorrhage, severe renal dysfunction, or compliance issues.

Intervention

- **Experimental Group:** Ticagrelor monotherapy (90 mg twice daily for 6 months).
- **Control Group:** Conventional DAPT (80 mg aspirin + 75 mg clopidogrel daily for 6 months).
- Antiplatelet therapy is initiated at or before PCI, with pre-stent loading doses.

Randomization and Allocation

- Randomization: 1:1 allocation via a web-based platform.
- Allocation concealment: Randomization code released only after patient recruitment.
- Randomization performed by the interventional cardiologist.

Blinding

- Neither the patients, investigators, nor data analysts will be aware of the group allocations. The tablets provided to participants are unmarked, and their characteristics and names will remain unknown throughout the study.
- Data entry and analysis are handled by a team separate from the research team to prevent bias.

Endpoints

Primary Efficacy Endpoint:

- Composite of cardiac death, target vessel myocardial infarction (MI), stent thrombosis, and need for revascularization post-PCI.

Secondary Efficacy Endpoints:

- All-cause death, major adverse cardiovascular events (MACE) including stroke, MI, arrhythmia, and individual components of the primary endpoint.

Primary Safety Endpoint:

- BARC (Bleeding Academic Research Consortium) bleeding type 3 or 5 post-PCI.

Secondary Safety Endpoint:

- BARC bleeding types 1-5.

Statistical Analysis

General Analysis:

- Continuous variables: Mean ± SD, analyzed using t-tests or Wilcoxon rank-sum tests.
- Categorical variables: Chi-square or Fisher's exact tests.
- Software: Stata (version 14.2).

Primary and Secondary Endpoint Analysis:

- Kaplan-Meier method for cumulative event rates.
- Cox log-rank test for time-to-event analysis.

Subgroup Analysis:

- Factors like age, sex, comorbidities, lesion characteristics, and procedural details.

Missing Data Handling:

- Patients lost to follow-up will be censored. Missing baseline variables will not be imputed.

Limitations

1. Single-center design may limit generalizability.
2. Potential challenges in ensuring participant compliance.

Flowchart

- A detailed visual representation of study processes is included below.

Outcome Assessment

- All events adjudicated by an independent clinical events committee.

Project Timeline

Step	Duration	Timeline (Months)
1. Proposal Completion and Ethics Approval	2 months	1 → 2
2. Registration on IRCT.ir and ClinicalTrials.gov	1 month	3
3. Collecting Baseline Data and Patient Recruitment	1 month	4
4. Implementation and Patient Follow-Up	8 months	5 → 12
5. Final Data Collection and Cleaning	1 month	13
6. Data Analysis and Results Compilation	1 month	14
7. Writing the Project Paper	1 month	15

Total Duration: 15 months

Detailed Timeline Representation (Months)

Step	1	2	3	4	5	6	7	8	9	10	11	12	13	14	15
Proposal and Ethics Approval	●	●													
Trial Registration			●												
Baseline Data Collection				●											
Patient Follow-Up and Trial					●	●	●	●	●	●	●	●			
Final Data Cleaning													●		
Data Analysis and Results														●	
Paper Writing															●

Report Submission Timeline

Report Title	Approximate Submission Time
**Progress Report**	End of Month 9
**Final Report (Publishable Format)**	End of Month 15

Personnel Costs

- **No data recorded.**

Specialized Tests and Services Costs

- **No data recorded.**

Equipment and Materials Costs

Item Name	Consumable/Non-consumable	Quantity	Unit	Unit Price (IRR)	Total (IRR)
**Ticagrelor**	Consumable	1,080	Package	109,710	118,486,800
**Total**					**118,486,800**

Travel Costs

- **No data recorded.**

Other Costs

- **No data recorded.**

Funding from External Organizations or University Grants

- **No data recorded**

Ethical Considerations

Ethical Approvals

1. **Research Ethics Approval:**
    - The protocol and informed consent templates will be reviewed and approved by the Institutional Review Board/Ethical Committee of Shiraz University of Medical Sciences.
2. **Protocol Amendments:**
    - Any significant changes to the protocol, including study objectives, design, population, sample size, or procedures, will require formal approval by the Ethical Committee/IRB of Shiraz University of Medical Sciences.

Consent and Confidentiality

1. **Consent Process:**
    - The trial will be introduced to patients by an interventional cardiologist.
    - Patients will receive detailed information sheets and will have the opportunity to discuss the trial with a consultant.
    - A trained general practitioner will obtain written informed consent from participants.
2. **Confidentiality Measures:**
    - All participant data will be stored securely in locked cabinets or password-protected systems.
    - Personal identifiers will be separated from study records and replaced with coded IDs to maintain confidentiality.
    - Only members of the Trial Management Committee (TMC) will have access to patient data and files.

Declaration of Interest

- The authors declare no conflicts of interest.
- The study does not involve sponsorship or funding agreements with pharmaceutical companies.
- Drugs (Aspirin, Clopidogrel, Ticagrelor) are prescribed from common and acceptable brands in Iran, without releasing specific brand names.

Post-Trial and Ancillary Care

- **Insurance Coverage:**
    - Shiraz University of Medical Sciences will provide insurance for harm caused by the protocol, including additional healthcare, compensation, or damages.
- **Post-Trial Care:**
    - The university will ensure appropriate follow-up for participants affected by the trial.

Commitment of the Investigator

- The investigator commits not to initiate the study before receiving ethics approval and an IRB code.
- If informed consent forms are required, the investigator will submit 10% of completed forms along with the trial's registration code in the IRCT system with the first progress report.

I hereby commit that I will not commence my study prior to obtaining approval and the ethical code from the Research Ethics Committee. If my study requires obtaining informed consent forms, I will submit 10% of the completed consent forms from patients/participants along with the study's registration code on the Iranian Clinical Trials website (IRCT) at the time of submitting the first progress report.

Informed Consent Form

1. Research Objectives:

To evaluate the efficacy and safety of ticagrelor compared to the combination of aspirin and clopidogrel in patients with chronic chest pain following percutaneous intervention (stent placement).

2. Participant Involvement in the Study:

Participants will be randomly assigned to two groups: one receiving ticagrelor monotherapy and the other receiving a combination of aspirin and clopidogrel post-stent placement.

Informed consent will be obtained before participation.

Blood samples will be taken, and patient data will be thoroughly reviewed.

After the intervention, participants will be evaluated monthly via phone calls and every two months in person for physician assessment until the end of the six-month period.

3. Blood Sampling:

A single blood sample will be taken before the intervention to conduct preliminary tests, including blood analysis, coagulation function, lipid profile, and electrolyte levels.

4. Potential Benefits:

Reduction in bleeding risks associated with aspirin use may be a potential benefit of this study.

5. Potential Risks:

An increased risk of vessel thrombosis may be a potential risk of the study.

6. Risk Mitigation:

Continuous monitoring and dedicated patient visits during the study period aim to minimize potential risks.

Immediate interventions will be performed if any risks arise.

7. Alternative Methods:

If the patient declines participation, the routine treatment with the combination of aspirin and clopidogrel will be initiated post-intervention.

8. Confidentiality:

Test results and study information will be kept confidential and reported only to the patient privately.

The researchers will remain blinded to patient identities and will only know the type of medication assigned to each participant without knowing their names.

9. Questions and Follow-Up:

Patients may direct any questions or follow-ups to the office of the primary investigator (Professor Kojuri) at:
Shiraz, Chamran, Niayesh Boulevard, Medical Building.
Phone: 071-36540068

Study Registration Code:

[To be provided upon registration]

Name:
Address:
Phone Number:
Signature:

Name:
Phone Number:
Signature:

Name:
Signature:

Commitment Form for Adherence to Ethical Principles in Medical Sciences Research and Compliance with Scientific Publication Laws Related to Student Theses

---

To: The Honorable Deputy of Research, Faculty of Medicine

I, **Professor Javad Kojouri**,
a faculty member of the **Cardiology Department**,
Faculty of Medicine,
as the primary supervisor of the thesis by **Mr./Ms. Davar Aldavood**,
a **Postdoctoral Fellowship** student,
entitled:

"Comparison of the Safety and Efficacy of Ticagrelor Monotherapy versus Standard Dual Antiplatelet Therapy in Chronic Coronary Syndrome Patients Post-PCI: A Single-Center, Randomized Clinical Trial,"

hereby confirm that all ethical principles in research will be adhered to at every stage of conducting this thesis. Furthermore, I will ensure compliance with scientific publication laws and authorship rights in the preparation and listing of authors for the resulting article, in accordance with relevant regulations.

Primary Supervisor's Name and Surname:
Professor Javad Kojuri

Date:
2023/12/26 (Persian Date: 1402/10/15)

Final Registration Commitment

I, on behalf of all individuals involved in or participating in this proposal, commit to thoroughly reviewing and adhering to all ethical considerations and guidelines for biomedical research ethics as outlined on the relevant Ethics Committees' websites of the Faculty/University. I further pledge to maintain adherence to these guidelines throughout the execution of this project and the submission of all resulting reports.

I, as the principal investigator of this study, confirm that this study has not previously been registered or submitted as a thesis or non-thesis project in any other center or faculty.

Scope:

Non-Communicable Diseases (NCDs)

Priority:

Basic and clinical studies (prevention, diagnosis, and treatment) in non-communicable diseases, with an emphasis on regional conditions.

Principal Investigator:

Yes, I agree.
